# Supplementary material for: Integrative computational approach identifies drug targets in CD4+ T-cell-mediated immune disorders
Source: NPJ Syst Biol Appl. 2021 Jan 22;7:4. doi: 10.1038/s41540-020-00165-3 (PMC7822845; doi:10.1038/s41540-020-00165-3)
Supplement: Supplementary file 1 — Compressed Supplementary Files [file 41540_2020_165_MOESM1_ESM.zip › Supplementary Data/Supplementary Information.pdf]

Integrative computational approach identifies drug targets in CD4+ T cell-mediated immune disorders

Bhanwar Lal Puniya, Rada Amin, Bailee Lichter, Robert Moore, Alex Ciurej, Sydney J. Bennett, Ab Rauf Shah, Matteo Barberis, Tomáš Helikar

Content

A. Supplementary Methods (Pages 2 - 19)

1. Pipeline for identification of potential metabolic targets of existing drugs
2. Development of novel genome-scale metabolic models of CD4+ T cells
3. Validation of metabolic models with literature and independent experimental data
4. Mapping drug targets
5. Gene expression analysis of autoimmune diseases
6. Perturbation of metabolic genes
7. Perturbation effect score (PES)
8. Selecting drug targets
9. Robustness and sensitivity of drug targets under different cutoffs
10. Software and tools used in this study
11. Supplementary References

B. Supplementary Tables (Page 20 - 23)

Supplementary Table 1: Metabolic models of CD4+ T cells

Supplementary Table 2: Identified CD4+ T cell drug targets for RA, MS, and PBC

C. Supplementary Figures (Pages 24 - 40)

Supplementary Figure 1: Characterizing expressed genes in CD4+ T cells

Supplementary Figure 2: Distribution of enzyme-catalyzed reactions across metabolic pathways

Supplementary Figure 3: Flux maps of metabolic pathways active in Th1 metabolic models

Supplementary Figure 4: Flux maps of metabolic pathways active in Th2 metabolic models

Supplementary Figure 5: Flux maps of metabolic pathways active in Th2 metabolic models

Supplementary Figure 6: Dependency of growth rate on Glucose in CD4T1670 model

Supplementary Figure 7: Lactate production by all the models

Supplementary Figure 8: Dependency of growth rate on glutamine in CD4T1670 model when glucose was removed.

Supplementary Figure 9: Accuracy and precision based on comparisons between essential genes predicted by models and experimentally defined

Supplementary Figure 10: Precision-recall curve of higher-ranked genes based effect on growth after inhibition (Naïve).

Supplementary Figure 11: Precision-recall curve of higher-ranked genes based effect on growth after inhibition (Th1).

Supplementary Figure 12: Precision-recall curve of higher-ranked genes based effect on growth after inhibition (Th2).

Supplementary Figure 13: Precision-recall curve of higher-ranked genes based effect on growth after inhibition (Th17).

Supplementary Figure 14: Distribution of PES and their Z-scores in different models

Supplementary Figure 15: CD4+ T cell proliferation response upon drug treatment

Supplementary Figure 16: Models constructed based on different datasets: transcriptomics, proteomics, and integrated

Supplementary Figure 17: Models constructed based on biomass reaction of iAB-AMO-1410 and Recon 3D

A. Supplementary Methods

1. Pipeline for identification of potential metabolic targets of existing drugs

In this work, we used an integrative computational approach that integrates experimental datasets, modeling, and literature mining. First, we constructed novel genome-scale metabolic models for four CD4+ T cell subtypes - naïve, Th1, Th2, and Th17. Second, we integrated existing compounds and drugs from The Repurposing Tool of ConnectivityMap (CMap)[84-85] database with the models. Third, we computationally inhibited existing drug targets affecting the reactions in naïve, Th1, Th2, and Th17 models, respectively. Next, the effects of perturbation on modeled metabolic reactions were characterized using flux ratios (flux under perturbed condition/ WT flux). Fourth, we identified differentially expressed genes (DEGs) associated with rheumatoid arthritis (RA), multiple sclerosis (MS), and primary biliary cholangitis (PBC) using available transcriptomics datasets. The identified DEGs were integrated with the metabolic models. Finally, we computed a score for each drug target based on its effect on the disease-associated DEGs. Based on this score, the drug targets were prioritized as potential treatment options for diseases. To further increase our confidence in the potential disease treatments, the identified drug targets were cross-referenced with literature.

2. Development of novel genome-scale metabolic models of CD4+ T cells

For metabolic model construction, we first characterized expressed genes in CD4+ T cells. Next, we use these genes with a template metabolic model to construct CD4+ T cell-specific models. Recon3D[35] was used as a template model, which was slightly modified prior to use.

2.1 Characterizing gene activities for CD4+ T cell subtypes

2.1.1 Data collection and analysis for characterizing gene activity for CD4+ T cell subtypes

2.1.1.1. Acquisition of data: The Gene Expression Omnibus (GEO)[69] database and PubMed were searched to obtain data for all five CD4+ T cell subtypes (naïve, Th1, Th2, and Th17). We searched for keywords such as “CD4+ T cells”, “CD4+ Th1”, “CD4+ Th2”, “CD4+ Th17” to obtain the data from GEO. Of all the datasets found, we retained the datasets for naïve CD4+ T cells (unpolarized), and the other subtypes where the naïve T cells were induced by cytokines to differentiate into one of the four T cell subtypes (Th1, Th2, and Th17). In these datasets, we selected all the datasets regardless of the time points when the samples were collected. Both unstimulated and stimulated cells were retained. In addition to that, both technical and biological replicates were kept. A total of 121 microarray samples were retained in the final list that includes gene expression series matrices GSE2770, GSE22886, GSE43769, GSE43005, GSE50175, GSE30664, GSE42569, GSE60679, GSE24634, and GSE22045 [22-30] (*Supplementary Data 1*). Additionally, we obtained one proteomics dataset from Rieckmann et al., 2017[31] from which 20 samples were used (*Supplementary Data 1*). For naïve CD4+ T cells, 8 samples were used, 4 samples for activated, and 4 samples are under steady-state. For other subtypes, 4 samples under steady-state conditions were used.

2.1.1.2. Analysis of the data: The gene expression datasets obtained from GEO were from different platforms i.e., Affymatrix, and Agilent. The datasets were analyzed in R mainly using *affy* and *limma* packages. The *Affymatrix* data was normalized using the MAS 5.0 normalization using the *affy* R package. Upon normalization, present, marginal, and absent (PMA) calls were generated. All the probes with P-value less than 0.05 were considered as expressed. *Agilent* datasets were quantile normalized and averaged over biological replicates. The proteomics dataset was obtained from a recently published study by (Rieckmann et al., 2017)[31]. All the proteins detected in three out of four samples were considered as expressed. The already processed proteomics data was used for analysis. Since we were interested in identifying either expressed or unexpressed genes, the continuous expression values in all the samples were converted into discrete values (0 and 1). For *Affymatrix* datasets, probes marked as present or marginal were considered expressed (1); probes marked as absent were considered unexpressed (0). For Agilent datasets, absence (0) and presence (1) of genes were determined as the genes which had higher expression levels than the negative control. The spots that were at least 40% brighter than the negative control were considered as expressed. For GSE43005, we used a cutoff that is 10% brighter than the negative control where 40% cutoff was very strict and resulted in much fewer genes. For each platform of gene expression, a consensus expressed call (1) across all samples for each probe was determined if at least half of the samples (in which the probe was present) showed the probe as expressed. A consensus unexpressed call (0) across all samples for each probe was determined if less than half of the samples showed the probe as unexpressed. For proteomic datasets, in each sample, an abundance greater than or equals to 1 meant that the protein was considered as present (1) and zero as absent (0). Similar to the microarray platforms, a consensus was made using all the samples whereby genes of measured proteins were considered expressed (1) if at least half of

the samples had a protein count greater than zero. Furthermore, to make all the datasets consistent and comparable, the accession ids in these datasets i.e., Affymetrix probe IDs, Agilent IDs were converted to Entrez IDs using the Ensembl BioMart package and corresponding platform packages in R.

2.1.1.3. Final expression matrix for genes (methods): We created three gene activity matrices for each cell type (1) based on the microarray data, (2) proteomics data, and (3) combining both microarray and proteomics datasets. The genes that were expressed were given value 1 and unexpressed as 0.

To create the combined activity matrix for genes expressed in T cells, we compared both microarray and proteomics datasets (Supplementary Figure 1a). The list of present genes (1) and then absent genes (0) from the microarray data and proteomics data were overlapped with one another. Present genes that overlapped from both microarray and proteomics data were considered expressed with high-confidence and were assigned a value of 1 for active. Absent genes that overlapped were considered unexpressed with high-confidence and were assigned a value of 0 for inactive. There are two possible reasons why there are genes that did not overlap from the two data pools: 1) only one dataset (microarray or proteomics) measured this particular gene, or 2) one dataset measured the gene present while the other measured it absent. To account for omitting false negatives and including false positives, further curation of data was conducted. Because of the high probability of omitting false negatives from the microarray data--due to the high number of gene IDs measured—non-overlapping gene IDs were considered expressed (1) if at least 90% of the samples for the given cell subtype showed the gene present. Non-overlapping gene IDs were considered unexpressed (0) if less than 10% of the samples for a given cell subtype showed the gene present. Moreover, the average protein count was calculated for four biological replicates for each cell subtype. Quartiles were

calculated using these averages, and gene IDs for proteins with counts within the top 25% expressed were considered active (1). Conversely, gene IDs for proteins with counts within the bottom 25% expressed were considered inactive (0). A two-column matrix was created for each subtype which included the Entrez IDs and their expressed or unexpressed call (*Supplementary data 10*).

Choosing the cutoff for Agilent datasets: GSE30664 and GSE60679 presented approximately 26000 and 40000 expressed probes, against 13000 for GSE43005. To filter out noise from GSE30664 and GSE60679, we used different cutoffs for the brightness over negative control and generated microarray consensus, and compared with proteomics data to confirm the results. GSE30664 consisted of two samples of Th1 and two samples of Th17 whereas GSE60679 contained 20 samples for Th17. For Th17, at 10% brightness cutoff, 41% of identified expressed genes from microarray datasets were confirmed from proteomics data. At 40% brightness cutoff, more than 50% of the expressed genes agreed with proteomics data. The effect was observed in Th17 because a high number of samples of Th17 come from Agilent data. Our results show that using a 40% cutoff significantly reduced the noise from the data. However, based on the number of probes expressed, a 40% cutoff becomes too strict for GSE435005 and results in only ~5000 probes expressed across all T cell subtypes as compared to ~12000 probes. Thus, we decided to use a 10% cutoff for GSE43005.

The comparison of genes identified as expressed from microarray data with a brightness cutoff of 10% for all three Agilent GSEs, 40% for three Agilent GSEs, and 40% cutoff for GSE30664 and GSE60679, and 10% for GSE435005 are shown in Table SM1. Results suggest 40% cutoff reduced the noise and overall improved the % overlap with proteomics data for Th17. Because of the low number of samples coming from the Agilent platform, naïve, Th1, and Th2 were not affected by these cutoffs.

Table SM1: Effect of different cutoffs on genes identified as expressed from Agilent microarray datasets

Brightness cutoff in Agilent Datasets	Number of expressed genes obtained based on consensus from Microarray datasets	Agreement with proteomics data	% agreement
10% brighter than negative control for GSE30664, GSE60679, and GSE435005	Naïve: 8367*<br>Th1: 10457<br>Th2: 9348*<br>Th17: 15478	Naïve: 4959*<br>Th1: 5759<br>Th2: 5461*<br>Th17: 6493	Naïve: 60%<br>Th1: 55%<br>Th2: 58%<br>Th17: 41%
40% brighter than negative control GSE30664, GSE60679, and GSE435005	Naïve: 7530<br>Th1: 10047<br>Th2: 9155<br>Th17: 11648	Naïve: 4532<br>Th1: 5634<br>Th2: 5368<br>Th17: 5894	Naïve: 60%<br>Th1: 56%<br>Th2: 59%<br>Th17: 51%
40% cutoff for (GSE30664 GSE60679)<br>and<br>10% cutoff for GSE435005	Naïve: 8367*<br>Th1: 10311<br>Th2: 9348*<br>Th17: 11666	Naïve:4959*<br>Th1: 5744<br>Th2:5461*<br>Th17: 5905	Naïve: 59%<br>Th1: 56%<br>Th2: 58%<br>Th17: 51%

*Highlighted samples should be identical because GSE30664 and GSE60679 do not include samples for naïve and Th2, thus changing the cutoff for these GSEs will not impact naïve and Th2. GSE43005 includes samples from all four cell subtypes.

Impact of changing GSE43005 cut-off on the final model: Next, we investigated the effect of choosing 40% brightness cutoffs for GSE43005 on identified expressed genes. Our result suggests that fewer than 1% of metabolic genes in the final list are changed, equivalent to 14, 7, and 2 metabolic genes in naïve, Th1, and Th2, respectively. No changes in expressed metabolic genes were observed for Th17.

We further investigated the effect of cutoff change in the models. In the naive model, out of 14 genes, 7 were included in the final version. The others were removed because they were

associated with blocked reactions. Of these seven (Entrez IDs: 54', '516', '8875', '57104', '91179', '130752', '160287') only one gene, ‘8875’ (R-Pantetheine Amidohydrolase), was critical (One reaction removed from the model if deleted). The other six genes included in the model have isozymes present; thus their deletion did not impact the model.

In Th1, out of seven genes, only two genes (Entrez IDs: ‘203’, ‘275’) were included in the final model. Of these two genes, only one gene, ‘275’, was critical, its deletion removing reactions from the glycine cleavage system.

In Th2, only two metabolic genes (Entrez IDs: ‘51660’, ‘79586’) were included because of the relaxed cutoff of GSE43005. Of these, the gene '51660' was critical and its deletion removed a reaction. This gene is involved in the Transport of Phenylpyruvate via Proton Symport (Mitochondrial).

Thus changing the cutoff from 10% to 40% will change one reaction in each model.

2.1.1.4 Final expression matrix for genes (Results): comparisons of expressed genes (from transcriptomics data) with genes of expressed proteins (in proteomics data) have shown a significant overlap (*Supplementary Figure 1a*). In all matched functions (genes or proteins) between transcriptomics and proteomics datasets, a total of 51.99%, 60.06%, 57.26%, and 60% functions were active in both datasets for naïve, Th1, Th2, and Th17 respectively. However, for these subtypes, significant proportions i.e., 72%, 80%, 77%, and 81% of total expressed proteins were common with expressed genes from the microarray datasets (*Supplementary Figure 1a*). To utilize the full potential of the data and increase the confidence in gene activity, we integrated the results from both microarray and proteomics datasets for Naïve, Th1, Th2, and Th17. This resulted in 6,942, 7,421, 6,671, and 9,042 genes that are expressed in naïve, Th1, Th2, and Th17 cells, respectively (*Supplementary Figure 1b*). A comparison of expressed genes among all the T cell subtypes resulted in 5,367 genes as commonly expressed

(*Supplementary Figure 1c*). Pathway analysis of the commonly expressed genes showed that biological processes related to the immune system were enriched (*Supplementary Figure 1d*). This includes gene ontology term *T cell receptor signaling pathway* (GO:0050852) enriched by 2.70 fold (110 genes, FDR < 1%). Similarly, KEGG pathways *T cell receptor signaling pathway* (hsa04660) was enriched by 1.78 fold (60 genes, FDR <1%).

2.1.2. Updates in the Recon3D model prior to use as a template

The most recent version of the Recon3D model was downloaded from (https://www.vmh.life) database. The MAT file obtained contains two models. We used the curated version of Recon3D that consists of 10,600 metabolic reactions. Prior to using Recon3D as a template for constructing metabolic models of CD4+ T cells, we made three types of changes in the model. These changes are as follows:

2.1.2.1. Updated GPR rules: GPR (gene protein reaction) association rules in the metabolic models are genes that encode for enzymes catalyzing reactions. These rules are written using the AND and *OR* operators. For example, if two hypothetical genes A and B encode for subunits of an enzyme the gene rule will be written as (A AND B). Similarly, if these two genes encode for two copies of the enzyme, their gene rule will be written as (A OR B). In Recon3D, transcripts ID's were used for GPR association rules. From transcriptomics and proteomics datasets we obtained genes expressed in CD4+ T cells (not transcript variants). We used the ENTREZ gene IDs in our data analysis. Thus, we converted transcript ID's in Recon 3D to ENTREZ gene IDs. The updated Recon3D comprises associations with 1,881 genes.

2.1.2.2. Removing reactions from Recon3D: During the model construction, we found four duplicated reactions (ARACHCRNt, C160CRNt, C180CRNt, TTDCRNt) in the template model that were removed.

2.1.2.3. Adding new reactions in Recon3D: New reactions added in the Recon3D model include a biomass reaction that was adopted from the human macrophage model iAB-AMØ-1410[74]. Other reactions were also added during the different iterations of the T cell subtype-specific model building. We added 13 new reactions in the model that are: PRPNCOAHYDm, EX_ca2[e], EX_cl[e], PPCOAOm, ARTPLM1, ARTPLM2, PIt2m, r0941, r1464, AIRCr_PRASCS, r0683, RTOT_3, ARTFR13. These reactions were obtained from Recon 2.2.05 and the macrophage model.

2.1.2.4. Media conditions: After these modifications, the final updated Recon 3D consists of 1,892 genes and 10,610 reactions. It is evident from the literature that CD4+ T cells have different preferences for nutrient uptake. Effector T cells are highly glycolytic and do not uptake fatty acids (FAs) from the environment whereas naïve T cells sense FAs (Supplementary References 1-2). Thus, the fatty acid exchange reactions were opened for the construction of naïve cells whereas they were closed for effector T cells. To capture that we created two template models each for naïve, and effector cells by constraining the exchange reactions of modified Recon3D. All the basic nutrients such as were set as unconstrained for all three template models. These media conditions were shown in Table SM1.

Table SM1: Open exchange reactions (media conditions) for two types of template models

Cell types	media conditions
**Naïve**	EX_ca2[e], EX_cl[e], EX_co[e], EX_fe2[e], EX_h[e], EX_h2o[e], EX_k[e], EX_na1[e], EX_nh4[e], EX_no2[e], EX_o2[e], EX_pi[e], EX_so4[e], EX_glc_D[e], EX_ala_L[e], EX_arg_L[e], EX_asn_L[e], EX_asp_L[e], EX_cys_L[e], EX_eicostet[e], EX_gln_L[e], EX_glu_L[e], EX_gly[e], EX_hdca[e], EX_hdcea[e], EX_his_L[e], EX_ile_L[e], EX_leu_L[e], EX_lnlc[e], EX_lnlnca[e], EX_lnlncg[e], EX_lys_L[e], EX_met_L[e], EX_ocdca[e], EX_ocdcea[e], EX_orn_D[e], EX_phe_L[e], EX_pro_L[e], EX_ribflv[e], EX_ser_L[e], EX_thr_L[e], EX_trp_L[e], EX_tyr_L[e], EX_val_L[e], EX_vitd3[e]
**Effector**	EX_ca2[e], EX_cl[e], EX_co2[e], EX_fe2[e], EX_h[e], EX_h2o[e], EX_k[e], EX_na1[e], EX_nh4[e], EX_no2[e], EX_pi[e], EX_so4[e], EX_glc_D[e], EX_o2[e], EX_ala_B[e], EX_ala_L[e], EX_arg_L[e], EX_asn_L[e], EX_asp_L[e], EX_cys_L[e], EX_gln_L[e], EX_glu_L[e], EX_gly[e], EX_his_L[e], EX_ile_L[e], EX_leu_L[e], EX_lys_L[e], EX_met_L[e], EX_orn[e], EX_orn_D[e], EX_phe_L[e], EX_pro_L[e], EX_ribflv[e], EX_ser_L[e], EX_thr_L[e], EX_trp_L[e], EX_tyr_L[e], EX_val_L[e], EX_vitd3[e]

2.1.2.5. Directionality changes: While refining the CD4+ T cell models (next section), the directionality of 95 reactions (shown below) was changed in Recon3D and rebuilt the models. [ADRNCPT1, ADRNCPT2, ADRNCRNt, ARACHCPT1, ARACHCPT2, C160CPT1, C160CPT2, C161CPT1, C161CPT12, C161CPT2, C161CPT22, C161CRN2t, C161CRNt, C180CPT1, C180CPT2, C181CPT1, C181CPT2, C181CRNt, C204CPT1, C204CPT2, C204CRNt, C226CPT1, C226CPT2, C226CRNt, C30CPT1, CLPNDCPT1, CLPNDCPT2, CLPNDCRNt, DCSPTN1CPT1, DCSPTN1CPT2, DCSPTN1CRNt, DLNLCGCPT1, DLNLCGCPT2, DLNLCGCRNt, EICOSTETCPT1, EICOSTETCPT2, EICOSTETCRNt, ELAIDCPT1, ELAIDCPT2, ELAIDCRNt, HEXDIACtr, HPDCACRNCPT1, HPDCACRNCPT2, HPDCACRNt, HXCOAc, HXCOAx, LNELDCCPT1, LNELDCCPT2, LNELDCCRNt, LNLCCPT1, LNLCCPT2, LNLCCRNt, LNLNCACPT1, LNLNCACPT2, LNLNCACRNt, LNLNCGCPT1, LNLNCGCPT2, LNLNCGCRNt, PCRNtm, PTDCACRNCPT1, PTDCACRNCPT2, PTDCACRNt, r0309, r0431, r0432, r0633, r0636, r0638, r0652, r0722, r0726, r0728, r0735, r0791, r1391, r1392, r1400, r1401, RE0583C, STRDNCCPT1, STRDNCCPT2, STRDNCCRNt, TETPENT3CPT1, TETPENT3CPT2, TETPENT3CRNt, TETPENT6CPT1, TETPENT6CPT2, TETPENT6CRNt, TETTET6CPT1, TETTET6CPT2, TETTET6CRNt, TMNDNCCPT1, TMNDNCCPT2, TMNDNCCRNt, TTDCPT2]

2.1.3. Construction of metabolic models of CD4+ T cells

We constructed metabolic models of CD4+ T cell subtypes using gene activities and a modified version of Recon3D.[35] In the modified Recon 3D model, we used a biomass equation of macrophage model iAB-AMØ-1410,[74] media conditions relevant to different CD4+ T cell subtypes. The GIMME method was used for model construction. Based on transcriptomics, proteomics, and integrated (multi-omics) data, we developed three cell-subtype specific

metabolic models for naïve, Th1, Th2, and Th17 cells. Models defined with the multi-omics data resulted in the lowest number of reactions (~ 37% to 49% of the modified Recon3D; Fig. 3a.). These models were significantly reduced because of strict cutoffs used in data integration resulted in only high-confidence expressed genes. Transcriptomics-based models had less number of reactions (~51 to 56% of modified Recon 3D) than proteomics-based models (~ 79% to 84% of modified Recon3D) because more transcriptomics studies led to strict cutoff than proteomics data. Because of the high confidence, we used multi-omics-based models of naïve, Th1, Th2, and Th17 for further analyses. In addition, we investigated how biomass objective functions in the template (modified Recon3D) model may affect the output models. We constructed new models with the same input data (final gene activity matrices) but with the original biomass objective function of Recon3D. In these models, more than 98% of reactions were common with models constructed with the iAB-AMØ-1410 biomass equation (*Supplementary Figure 2*). Thus, no significant changes in the constructed cell-type-specific models were observed based on these two objective functions in the template model. Based on the macrophage biomass equation our models obtained better flux distribution through fatty acid metabolic pathways. Thus, we selected final subtype-specific models that are based on the iAB-AMØ-1410 biomass equation (*Supplementary Data 3 - 4*). These final metabolic models have more genes than the number of expressed genes identified in the data. That is, if a reaction is active, the entire gene rule was retained in the model by the Gene Inactivity Moderated by Metabolism and Expression (GIMME) algorithm (Becker and Palsson, 2008). Furthermore, the GIMME method requires an objective function and favors reinsertion of removed reactions that are needed for Required Metabolic Functionalities (RMFs) even if those are not supported by the data.

Additionally, during model construction and refinement, the directionality of many reactions was changed based on data obtained from Recon 2.2.05[73] and MetaCyc database (https://metacyc.org/) (Supplementary Reference: 3). In the updated Recon3D, model a total of 95 reactions were changed from reversible to irreversible (See the previous section). In each final CD4+ T cell model, these reactions are shown in Table SM2.

Table SM2: Directionality of reactions changed during model refinement. Reactions in final T cell models with changed directionality from Recon3D are shown.

CD4+ T cell subtypes	Directionality changed (Reversible to irreversible)
**Naïve**	TTDCPT2, TMNDNCCRNt, TMNDNCCPT2, TMNDNCCPT1, TETTET6CRNt, TETTET6CPT2, TETTET6CPT1, TETPENT6CRNt, TETPENT6CPT2, TETPENT6CPT1, TETPENT3CRNt, TETPENT3CPT2, TETPENT3CPT1, STRDNCCRNt, STRDNCCPT2, STRDNCCPT1, RE0583C, r1401, r1400, r0791, r0735, r0652, r0638, r0636, r0633, r0432, r0431, r0309, PTDCACRNt, PTDCACRNCPT2, PTDCACRNCPT1, PCRNtm, LNLNCGCRNt, LNLNCGCPT2, LNLNCGCPT1, LNLNCACRNt, LNLNCACPT2, LNLNCACPT1, LNLCCRNt, LNLCCPT2, LNLCCPT1, LNELDCCRNt, LNELDCCPT2, LNELDCCPT1, HXCOAx, HXCOAc, HPDCACRNt, HPDCACRNCPT2, HPDCACRNCPT1, HEXDIACtr, ELAIDCRNt, ELAIDCPT2, ELAIDCPT1, EICOSTETCRNt, EICOSTETCPT2, EICOSTETCPT1, DLNLCGCRNt, DLNLCGCPT2, DLNLCGCPT1, DCSPTN1CRNt, DCSPTN1CPT2, DCSPTN1CPT1, CLPNDCRNt, CLPNDCPT2, CLPNDCPT1, C30CPT1, C226CRNt, C226CPT2, C226CPT1, C204CRNt, C204CPT2, C204CPT1, C181CRNt, C181CPT2, C181CPT1, C180CPT2, C180CPT1, C161CRNt, C161CRN2t, C161CPT22, C161CPT2, C161CPT12, C161CPT1, C160CPT2, C160CPT1, ARACHCPT2, ARACHCPT1, ADRNCRNt, ADRNCPT2, ADRNCPT1
**Th1**	ADRNCPT2, ADRNCRNt, C160CPT2, C161CPT2, C161CPT22, C161CRN2t, C161CRNt, C180CPT2, C181CPT2, C181CRNt, C204CPT2, C204CRNt, C226CPT2, C226CRNt, CLPNDCPT2, CLPNDCRNt, DCSPTN1CPT2, DCSPTN1CRNt, DLNLCGCPT2, DLNLCGCRNt, EICOSTETCPT2, EICOSTETCRNt, ELAIDCPT2, ELAIDCRNt, HPDCACRNCPT2, HPDCACRNt, HXCOAc, HXCOAx, LNELDCCPT2, LNELDCCRNt, LNLCCPT2, LNLCCRNt, LNLNCGCPT2, LNLNCGCRNt, PCRNtm, PTDCACRNCPT2, PTDCACRNt, r0309, r0633, r0636, r0638, r0652, r0735, r1400, r1401, RE0583C, STRDNCCPT2, STRDNCCRNt, TMNDNCCPT2, TMNDNCCRNt, TTDCPT2
**Th2**	TTDCPT2, TMNDNCCRNt, TMNDNCCPT2, TMNDNCCPT1, TETTET6CRNt, TETTET6CPT2, TETTET6CPT1, TETPENT6CRNt, TETPENT6CPT2, TETPENT6CPT1, TETPENT3CRNt, TETPENT3CPT2, TETPENT3CPT1, STRDNCCRNt, STRDNCCPT2, STRDNCCPT1, RE0583C, r1401, r1400, r0791, r0735, r0652, r0638, r0636, r0633, r0432, r0431, r0309, PTDCACRNt, PTDCACRNCPT2, PTDCACRNCPT1, PCRNtm, LNLNCGCRNt, LNLNCGCPT2, LNLNCGCPT1, LNLNCACRNt, LNLNCACPT2, LNLNCACPT1, LNLCCRNt, LNLCCPT2, LNLCCPT1, LNELDCCRNt, LNELDCCPT2, LNELDCCPT1, HXCOAx, HXCOAc, HPDCACRNt, HPDCACRNCPT2, HPDCACRNCPT1, HEXDIACtr, ELAIDCRNt, ELAIDCPT2, ELAIDCPT1, EICOSTETCRNt, EICOSTETCPT2, EICOSTETCPT1, DLNLCGCRNt, DLNLCGCPT2, DLNLCGCPT1, DCSPTN1CRNt, DCSPTN1CPT2, DCSPTN1CPT1, CLPNDCRNt, CLPNDCPT2, CLPNDCPT1, C30CPT1, C226CRNt, C226CPT2, C226CPT1, C204CRNt, C204CPT2, C204CPT1, C181CRNt, C181CPT2, C181CPT1, C180CPT2, C180CPT1, C161CRNt, C161CRN2t, C161CPT22, C161CPT2, C161CPT12, C161CPT1,

	C160CPT2, C160CPT1, ARACHCPT2, ARACHCPT1, ADRNCRNt, ADRNCPT2, ADRNCPT1
**Th17**	ADRNCPT1, ADRNCPT2, ADRNCRNt, ARACHCPT1, ARACHCPT2, C160CPT1, C160CPT2, C161CPT1, C161CPT12, C161CPT2, C161CPT22, C161CRN2t, C161CRNt, C180CPT1, C180CPT2, C181CPT1, C181CPT2, C181CRNt, C204CPT1, C204CPT2, C204CRNt, C226CPT1, C226CPT2, C226CRNt, C30CPT1, CLPNDCPT1, CLPNDCPT2, CLPNDCRNt, DCSPTN1CPT1, DCSPTN1CPT2, DCSPTN1CRNt, DLNLCGCPT1, DLNLCGCPT2, DLNLCGCRNt, EICOSTETCPT1, EICOSTETCPT2, EICOSTETCRNt, ELAIDCPT1, ELAIDCPT2, ELAIDCRNt, HEXDIACtr, HPDCACRNCPT1, HPDCACRNCPT2, HPDCACRNt, HXCOAc, HXCOAx, LNELDCCPT1, LNELDCCPT2, LNELDCCRNt, LNLCCPT1, LNLCCPT2, LNLCCRNt, LNLNCACPT1, LNLNCACPT2, LNLNCACRNt, LNLNCGCPT1, LNLNCGCPT2, LNLNCGCRNt, PCRNtm, PTDCACRNCPT1, PTDCACRNCPT2, PTDCACRNt, r0309, r0431, r0432, r0633, r0636, r0638, r0652, r0735, r0791, r1400, r1401, RE0583C, STRDNCCPT1, STRDNCCPT2, STRDNCCRNt, TETPENT3CPT1, TETPENT3CPT2, TETPENT3CRNt, TETPENT6CPT1, TETPENT6CPT2, TETPENT6CRNt, TETTET6CPT1, TETTET6CPT2, TETTET6CRNt, TMNDNCCPT1, TMNDNCCPT2, TMNDNCCRNt, TTDCPT2

3. Validation of metabolic models with literature and independent experimental data

Pathway- and gene-essentiality-based validations of models were performed. In addition to the validations presented in the results section of the manuscript, we tested T-cell specific essential genes: glutamine synthetase (GLNS) and acetyl-CoA carboxylase (ACC1) that were experimentally observed as essential for CD4+ T cell function. We specifically inhibited ACC1 and GLNS in all the models and simulated their effect on the growth rate. In our models, knocking out GLNS does not have any effect. However, knocking out GLNS in the absence of extracellular Glutamine will completely block the growth in effector T cells, suggesting a partial agreement with the literature. Similarly, our models identified ACC1 as essential for Th1, Th2, and Th17. These observations are indeed in agreement with the literature (see *Materials and Methods*) suggesting that blocking ACC1 inhibits Th17 cells.

4. Mapping drug targets

The existing drug target genes and their annotations were exported from The Drug Repurposing Hub tool in the ConnectivityMap (CMap) database (https://clue.io/repurposing). The exported

table contained the drugs, their targets, and their mechanism of action. From this table, we first removed all the withdrawn drugs and the next selected drugs from the class inhibitors. The gene symbols in the table were converted to Entrez IDs that were mapped in the metabolic models.

5. Gene expression analysis of autoimmune diseases

To identify genes that are differentially expressed in autoimmune diseases, we searched the GEO database. We searched for the datasets that were obtained from CD4+ T cells from patients with autoimmune diseases. A total of three datasets GSE56649, GSE43591, and GSE93170 were found in which the samples were extracted from peripheral CD4+ T cells. GSE56649 contains expression data from 13 active rheumatoid arthritis patients and nine healthy controls. GSE43591 contains the data from 10 patients with multiple sclerosis and 10 healthy controls. GSE93170 consists of data from six patients with primary biliary cholangitis and six healthy controls. The raw files of GSE56649 and GSE43591 were processed using the *affy* package in Bioconductor. The GSE93170 was processed with the *limma* package. We used the *limma* package for differential gene expression.

6. Perturbation of metabolic genes

The knockout of genes was performed in MATLAB using the COBRA toolbox. MoMA method in the *singleGeneDeletion* function was used to compute fluxes under gene knockout conditions. Based on these analyses, we first identified genes that blocked reaction(s) in the model using *hasEffect* output (of the *singleGeneDeletion* function) that returned TRUE. The flux solution under these gene knockouts was selected. Next, flux obtained under each gene deletion was compared with wild type (without any knockout) flux obtained by *OptimizeCbModel* function,

which resulted in the flux ratio for each gene. For each model, flux ratios under each gene deletion were combined in a table.

7. Perturbation effect score (PES)

The PES was calculated for each gene that was perturbed in models. The PES was calculated for all models and disease combinations. We used the following steps for PES calculation:

A. Using flux ratios obtained for each gene knockout, we selected reactions that are associated with genes that were differentially expressed in a disease.
B. From selected reactions, we removed reactions that had zero flux in both KO and WT conditions (that produced NaN in the flux ratio table).
C. We counted fluxes that were increased and decreased. The flux was considered as increased if the flux ratio was > 1 and decreased when the flux ratio was < 0.99. If the flux ratio value was zero it was considered as decreased. Flux ratios that had the "Infinity" value were considered as increased.
D. We counted unique genes within the following six terms: (i) Fluxes controlled by genes upregulated in the disease that were decreased after knockout (UpDec). (ii) Fluxes controlled by genes downregulated in disease but increased after knockout (UpInc). (iii) Fluxes controlled genes upregulated in disease but were unchanged after gene knockout (UpUnc). (iv) Fluxes controlled by genes downregulated in diseases but increased after knockout (DownInc), (v) fluxes controlled by genes downregulated in disease but decreased after perturbation of a gene (DownDec), (vi) fluxes controlled by genes downregulated in disease but unchanged after knockout (DOwnUnc).
E. We counted unique genes within each term and used them in the formula of PES given in the Materials and Methods.

F. PES for genes were calculated using all models in three diseases (4 models X 3 disease = 12 combinations).

8. Selecting drug targets

Within each model, the PES was first transformed into ranks by sorting them in descending order. Next, these ranks were used to calculate Z-scores. From each model, the mean (μ) and standard deviation (σ) of PES ranks were calculated. x is the PES rank in a model.

$$Z - score\ =\ \frac{(x-\mu)}{\sigma}$$

Our next aim was to identify better drug targets by using all the Z-scores. To combine ranks, we summed up four Z-scores of models and obtained aggregated Z-score. The genes were ranked by sorting aggregated Z-scores in ascending order. Genes with aggregated Z-score -1 were selected as potential candidates for drug targets.

9. Robustness and sensitivity of drug targets under different cutoffs

We investigated the sensitivity and robustness of drug targets to cutoffs used in this study. Using a 1.5-fold and adjusted P-value < 0.05 cutoff for differential gene expression led us to identify 27 drug targets for RA. Both 1.5-fold and 2-fold cutoffs (with adjusted P-value < 0.05) identified nine common genes (ABAT, NAMPT, EPHX2, PDHB, COMT, HIBCH, SOD2, GAA, ACAT1). MS and PBC had zero differentially expressed genes at 2-fold cutoff; thus, we used a minimum acceptable cutoff of 1.5 and adjusted P-value < 0.05.

Furthermore, we also investigated if the identified drug targets were sensitive to the glucose uptake rates used in the model. We found two drug targets for RA (HIBCH, SOD2), eight for MS

(DPYD, IDH2, GSR, PKM, ABAT, LSS, FASN, SQLE), and two for PBC (APRT, TXNRD1) that were sensitive to glucose uptake rates.

10. Software and databases used in this work

Table SM3: URLs of software and databases used in this work

Tool type	Task	Name of the tool	URL
Software	Gene Expression Data Analysis	R-3.4.3	https://www.r-project.org/
		Affy R-package	https://www.bioconductor.org/packages/release/bioc/html/affy.html
		Limma R-package	http://bioconductor.org/packages/release/bioc/html/limma.html
	Model construction and analysis	MATLAB R2016b	https://www.mathworks.com/?s_tid=gn_logo
		COBRA toolbox (Matlab)	https://opencobra.github.io/cobratoolbox/stable/
Databases	Model Refinement	MetaCyc	https://metacyc.org/
	Gene Essentiality Analysis	OGEE database	http://ogee.medgenius.info/browse/
	Gene expression Data mining	GEO database	https://www.ncbi.nlm.nih.gov/geo/
	Gene Functions	UniProt	https://www.uniprot.org/
		GeneCards	https://www.genecards.org/
	Pathway enrichment analysis	DAVID	https://david.ncifcrf.gov/
		STRING	https://string-db.org/
		KEGG pathways	https://www.genome.jp/kegg/pathway.html
		Gene Ontology	http://geneontology.org/
		Reactome	https://reactome.org/
	Drug Repurposing	CMap database	https://clue.io/cmap
	Drug Targets	ChEMBL	https://www.ebi.ac.uk/chembl/

		DrugBank	https://www.drugbank.ca/
	Literature Mining	PubMed	https://www.ncbi.nlm.nih.gov/pubmed/

11. Supplementary References

1. Kedia-Mehta, N. & Finlay, D. K. Competition for nutrients and its role in controlling immune responses. Nat. Commun. 10, 2123 (2019).
2. Wei, J., Raynor, J., Nguyen, T.-L. M. & Chi, H. Nutrient and Metabolic Sensing in T Cell Responses. Front. Immunol. 8, (2017).
3. Caspi, R. *et al.* The MetaCyc database of metabolic pathways and enzymes and the BioCyc collection of Pathway/Genome Databases. *Nucleic Acids Res.* 42, D459–D471 (2014).

B. Supplementary Tables

Supplementary Table 1: Metabolic models of CD4+ T cells

	**Naïve**	**Th1**	**Th2**	**Th17**	**CD4T1670 (Naïve) (reduced)***
**Genes**	1055	1133	1127	1250	1027
**Reactions**	5179	3956	5252	5282	2592
**Internal reactions (Enzyme catalyzed)**	2501	1969	2549	2640	1146
**Metabolites**	3153	2517	3156	3263	1402

* Our models do not include dead ends and use gene IDs. To make CD4T1670 comparable to our models, we removed dead ends and counted the number of genes in the reduced model (in place of transcripts).

Supplementary Table 2: Identified CD4+ T cell drug targets for RA, MS, and PBC

Disease	Entrez ID	Gene Symbol	Gene description	Aggregate Z-score	Experimental evidences relevant to CD4+ T cells and autoimmune diseases	ChEMBL [96]IDs*
RA	4047	LSS	Lanosterol synthase	-3.35	Inhibition of lanosterol synthase (LSS) might decrease the endogenous cholesterol that may lead to impact cell division. [97]	CHEMBL3593
	18	ABAT	4-aminobutyrate aminotransferase	-3.20	GABA downregulate inflammatory response in a mouse model of RA; [42] Inhibition of ABAT might increase GABA.[16]	CHEMBL2044
	10135	NAMPT	Nicotinamide phosphoribosyltransferase	-3.11	Nampt inhibition reduces demyelination and disability in EAE[56]), Lack of NAMPT expression affect T cell development.[98]	CHEMBL1744525
	2224	FDPS	Farnesyl pyrophosphate synthase	-2.98	Inhibition of FDPS inhibit T cell cytokine production.[99]	CHEMBL1782
	6713	SQLE	Squalene monooxygenase	-2.96	Increased Membrane Cholesterol in T cells leads to inflammatory response. [100]	CHEMBL3592
	2222	FDFT1	Farnesyl-diphosphate farnesyltransferase	-2.66	No support	CHEMBL3338
	2053	EPHX2	Bifunctional epoxide hydrolase 2	-2.44	Inhibition of EPHX2 pre clinically evaluated as drug target for IBD.[58]	CHEMBL2409
	4967	OGDH	2-oxoglutarate dehydrogenase	-2.30	No support	CHEMBL2816
	847	CAT	Catalase	-2.22	Protect T cells against oxidative stress.[101]	CHEMBL3627594
	1431	CS	Citrate synthase	-2.17	Inhibition of citrate synthase leads to reduction in citrate leading to reduced proliferation.[102]	DB02637
	5162	PDHB	Pyruvate dehydrogenase E1 component subunit beta	-2.04	**No inhibition of CD4+ T cell proliferation under treatment with drug ethyl-pyruvate (This study)**	DB00119
	1312	COMT	Catechol O-methyltransferase	-1.80	**Reduced CD4+ T cell proliferation when inhibited using drug entacapone (This study)**	CHEMBL2023
	26275	HIBCH	3-hydroxyisobutyryl-CoA hydrolase	-1.73	**No inhibition of CD4+ T cell proliferation under treatment with drug quercetin (This study)**	CHEMBL3817723
	6648	SOD2	Superoxide dismutase [Mn], mitochondrial	-1.32	Loss of SOD2 increased superoxide, and defective T cell development.[62]	CHEMBL4105776
	1723	DHODH	Dihydroorotate dehydrogenase	-1.26	Explored as a potential drug target for RA[43] and MS[103].	CHEMBL1966
	2548	GAA	alpha-glucosidase	-1.06	No support	CHEMBL2608
	38	ACAT1	Acetyl-CoA acetyltransferase, mitochondrial	-1.01	Target of Sulfasalazine that is anti inflammatory indicated for treatment of ulcerative colitis and rheumatoid arthritis.[50]	CHEMBL2616
MS	1376	CPT2	Carnitine O-palmitoyltransferase 2	-3.09	**Reduced CD4+ T cell proliferation when inhibited using drug perhexiline (This study)**	CHEMBL3238
	847	CAT	Catalase	-3.08	Protect T cells against oxidative stress.[101]	CHEMBL3627594
	498	ATP5F1A	ATP synthase subunit alpha	-3.04	**No inhibition of CD4+ T cell proliferation under treatment with drug quercetin (This study)**	CHEMBL2062351
	506	ATP5F1B	ATP synthase subunit beta	-2.88	**No inhibition of CD4+ T cell proliferation under treatment with drug quercetin (This study)**	CHEMBL2062350
	509	ATP5F1C	ATP synthase F1 subunit gamma	-2.71	**No inhibition of CD4+ T cell proliferation under treatment with drug quercetin (This study)**	DB04216
	4953	ODC1	Ornithine decarboxylase	-2.67	**No inhibition of CD4+ T cell proliferation under treatment with drug DFMO (This study)**	CHEMBL1869
	471	ATIC	MP cyclohydrolase	-2.66	**No inhibition of CD4+ T cell proliferation under treatment with drug pemetrexed(This study)**	CHEMBL3430882
	513	ATP5F1D	ATP synthase subunit delta	-2.55	No support	DB00228
	515	ATP5PB	ATP synthase F(0) complex subunit B1	-2.39	No support	BTB06584 (cMap)
	128	ADH5	Alcohol dehydrogenase class-3	-2.19	**No inhibition of CD4+ T cell proliferation under treatment with compound N6022 (This study)**	CHEMBL4116
	1806	DPYD	Dihydropyrimidine dehydrogenase	-1.83	**Reduced CD4+ T cell proliferation when inhibited using drug perhexiline (This study)**	CHEMBL3172
	3418	IDH2	Isocitrate dehydrogenase	-1.61	knockdown of IDHI or IDH2 reduces IL-17 producing cells (Patent WO2017123808A1). [104]	CHEMBL3991501

	2936	GSR	Glutathione reductase	-1.59	Inhibition of GSH de novo synthesis reduces the pathological progression of EAE. [44]	DB0262
	3156	HMGCR	3-hydroxy-3-methylglutaryl-coenzyme A reductase	-1.58	Potential target for autoimmune diseases. [105]	CHEMBL402
	2222	FDFT1	Farnesyl-diphosphate farnesyltransferase	-1.55	No support	CHEMBL3338
	1719	DHFR	Dihydrofolate reductase	-1.52	Low dose Methotrexate (inhibitor of DHFR) found effective for MS, RA, and Crohn's disease. [45]	CHEMBL202
	5315	PKM	Pyruvate kinase	-1.51	Potential target to regulate inflammation. [59]	CHEMBL1075189
	2618	GART	phosphoribosylglycinamide formyltransferase	-1.46	**No inhibition of CD4+ T cell proliferation under treatment with drug pemetrexed (This study)**	CHEMBL3972
	18	ABAT	4-aminobutyrate aminotransferase	-1.41	GABA downregulates inflammatory response in a mouse model of RA; [42] Inhibition of ABAT might increase GABA. [16]	CHEMBL2044
	4047	LSS	Lanosterol synthase	-1.28	Inhibition of lanosterol synthase (LSS) might decrease the endogenous cholesterol, leading to impact cell division. [97]	CHEMBL3593
	2194	FASN	Fatty acid synthase	-1.16	Fatty acid synthase linked to the pathogenicity of Th17 cells. [106]	CHEMBL4106134
	5471	PPAT	phosphoribosyl pyrophosphate amidotransferase	-1.15	Knockdown of CAD and PPAT promotes regulatory CD4+ T cells.[107]	CHEMBL2362992
	4860	PNP	Purine nucleoside phosphorylase	-1.12	inhibition leads to T cell suppression.[108]	CHEMBL4338
	1431	CS	Citrate synthase	-1.11	Increased Citrate in MS patients.[109]	DB02637
	293	SLC25A6	ADP/ATP translocase 3	-1.11	No support	CHEMBL4105854
	790	CAD	carbamoyl-phosphate synthetase 2	-1.03	Knockdown of CAD and PPAT promotes regulatory CD4+ T cells.[107]	CHEMBL3093
	6713	SQLE	Squalene monooxygenase	-1.00	Increased Membrane Cholesterol in T cells leads to an inflammatory response. [100]	CHEMBL3592
PBC	10135	NAMPT	Nicotinamide phosphoribosyltransferase	-6.10	Nampt inhibition reduces demyelination and disability in EAE [56], Lack of NAMPT expression affect T cell development. [98]	CHEMBL1744525
	3704	ITPA	Inosine triphosphate pyrophosphatase	-5.31	No support	CHEMBL4105788
	132	ADK	Adenosine kinase	-4.90	No support	CHEMBL3589
	2181	ACSL3	Long-chain-fatty-acid--CoA ligase 3	-4.42	**Reduced CD4+ T cell proliferation when inhibited using drug EPA (This study)**	DB00159
	1890	TYMP	Thymidine phosphorylase	-4.13	No support	CHEMBL3106
	262	AMD1	S-adenosylmethionine decarboxylase proenzyme	-4.04	No support	CHEMBL4181
	353	APRT	Adenine phosphoribosyltransferase	-3.89	No support	CHEMBL4105819
	128	ADH5	Alcohol dehydrogenase class-3	-3.89	No support	CHEMBL4116
	1312	COMT	Catechol O-methyltransferase	-3.71	**Reduced CD4+ T cell proliferation when inhibited using drug entacapone (This study)**	CHEMBL2023
	2053	EPHX2	Bifunctional epoxide hydrolase 2	-3.64	Inhibition of EPHX2 pre clinically evaluated as drug target for IBD.[58]	CHEMBL2409
	26275	HIBCH	3-hydroxyisobutyryl-CoA hydrolase	-3.64	**No inhibition of CD4+ T cell proliferation under treatment with drug quercetin (This study)**	CHEMBL3817723
	2194	FASN	Fatty acid synthase	-3.62	Fatty acid synthase linked to the pathogenicity of Th17 cells. [106]	CHEMBL4106134
	2720	GLB1	Beta-galactosidase	-3.56	No support	CHEMBL2522
	114971	PTPMT1	Phosphatidylglycerophosphatase and protein-tyrosine phosphatase 1	-3.28	No support	CHEMBL2052033
	100	ADA	Adenosine deaminase	-3.15	ADA is a potential target for the treatment of inflammatory disorders. [110]	CHEMBL1910
	2739	GLO1	Lactoylglutathione lyase	-3.10	No support	CHEMBL2424
	2539	G6PD	Glucose-6-phosphate 1-dehydrogenase	-3.06	No support	CHEMBL5347

	3251	HPRT1	Hypoxanthine-guanine phosphoribosyltransferase	-2.97	No support	CHEMBL3243916
	2548	GAA	alpha-glucosidase	-2.96	No support	CHEMBL2608
	6515	SLC2A3	Solute carrier family 2, facilitated glucose transporter member 3	-2.51	Glut3 expressed in differentiated cells and resting equals to glut1.[37]	CHEMBL5215
	4363	ABCC1	Multidrug resistance-associated protein 1	-2.31	No support	CHEMBL3004
	7296	TXNRD1	Thioredoxin reductase 1	-1.99	essential for DNA synthesis during T-cell metabolic reprogramming and proliferation.[111]	CHEMBL1927
	6647	SOD1	Superoxide dismutase	-1.67	No support	CHEMBL2354
	47	ACLY	ATP-citrate synthase	-1.57	Inactivation of ACLY reduces IL-2-promoted CD4+ T-cell growth.[112]	CHEMBL3720

*When ChEMBL ids were not available, the DrugBank[50] ids or names of the molecules are provided as given in the repurposing tool of the cMap database.

Descriptions of drug targets tested in this study are shown in bold.

C. Supplementary Figures

Supplementary Figure 1: Characterizing expressed genes in CD4+ T cells

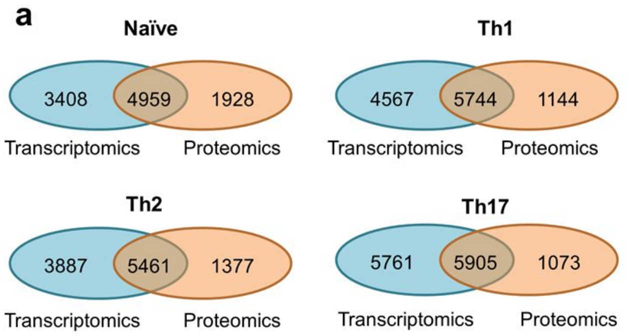

b

T cell subtypes	No. of expressed genes (Transcriptomics)	No. of expressed genes (proteomics)	No. of expressed genes (integrated data)
Naive	8367	6887	6942
Th1	10311	6888	7421
Th2	9348	6838	6671
Th17	11666	6978	9042

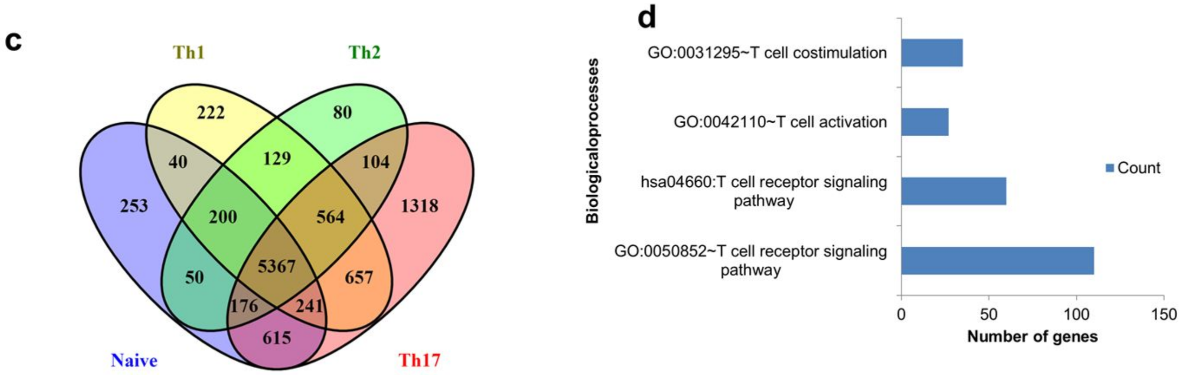

(a) Venn diagrams show a comparison between expressed genes identified in transcriptomics and proteomics data. (b) Table showing the total number of expressed genes from transcriptomics, proteomics, and integrated transcriptomics and proteomics datasets. (c) A comparison of expressed genes among four CD4+ T cell subtypes. (d) Enriched T cell-specificbiological processes in genes expressed in all subtypes.

Supplementary Figure 2: Distribution of enzyme-catalyzed reactions across metabolic pathways

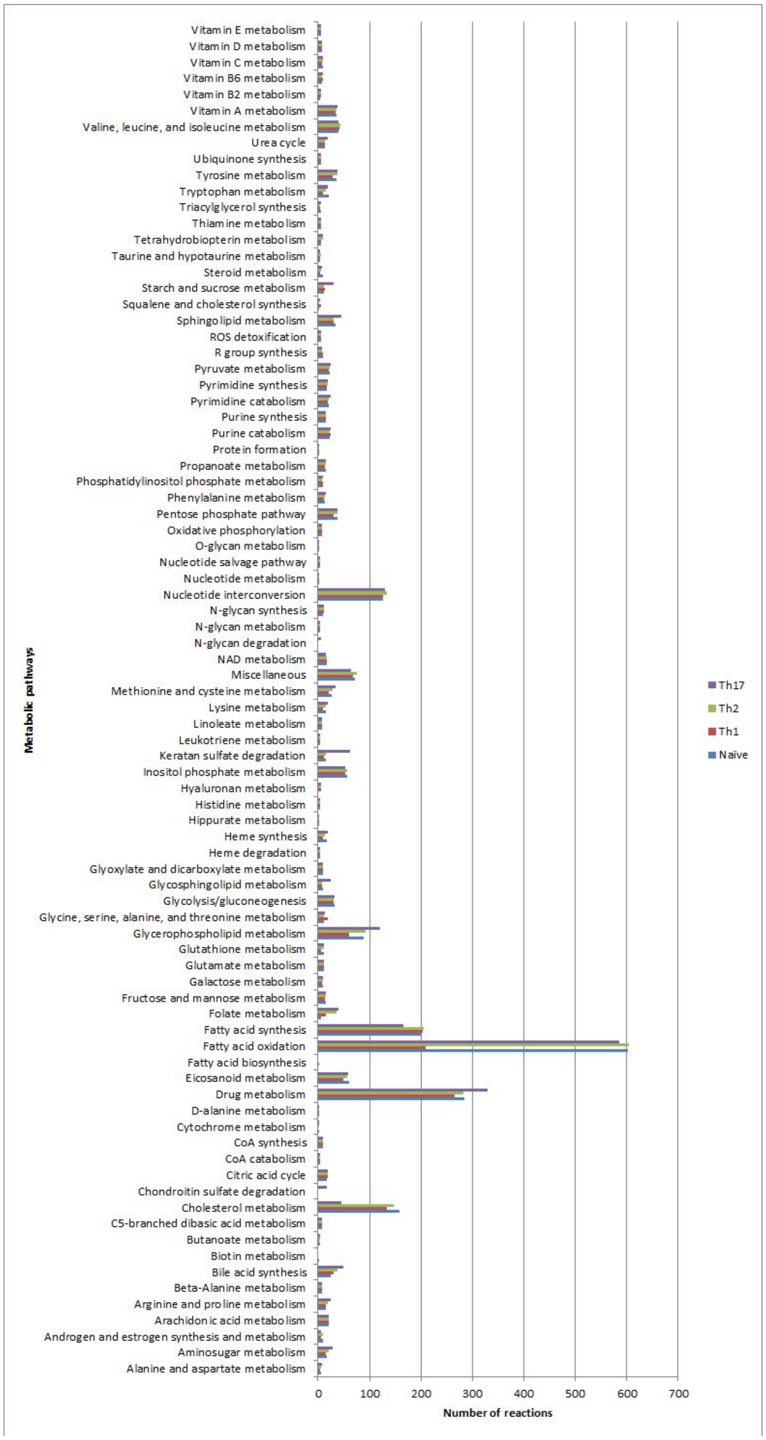

The figure shows the number of reactions distributed across 84 metabolic pathways in five metabolic models of CD4+ T cells

Supplementary Figure 3: Flux maps of metabolic pathways active in Th1 metabolic models

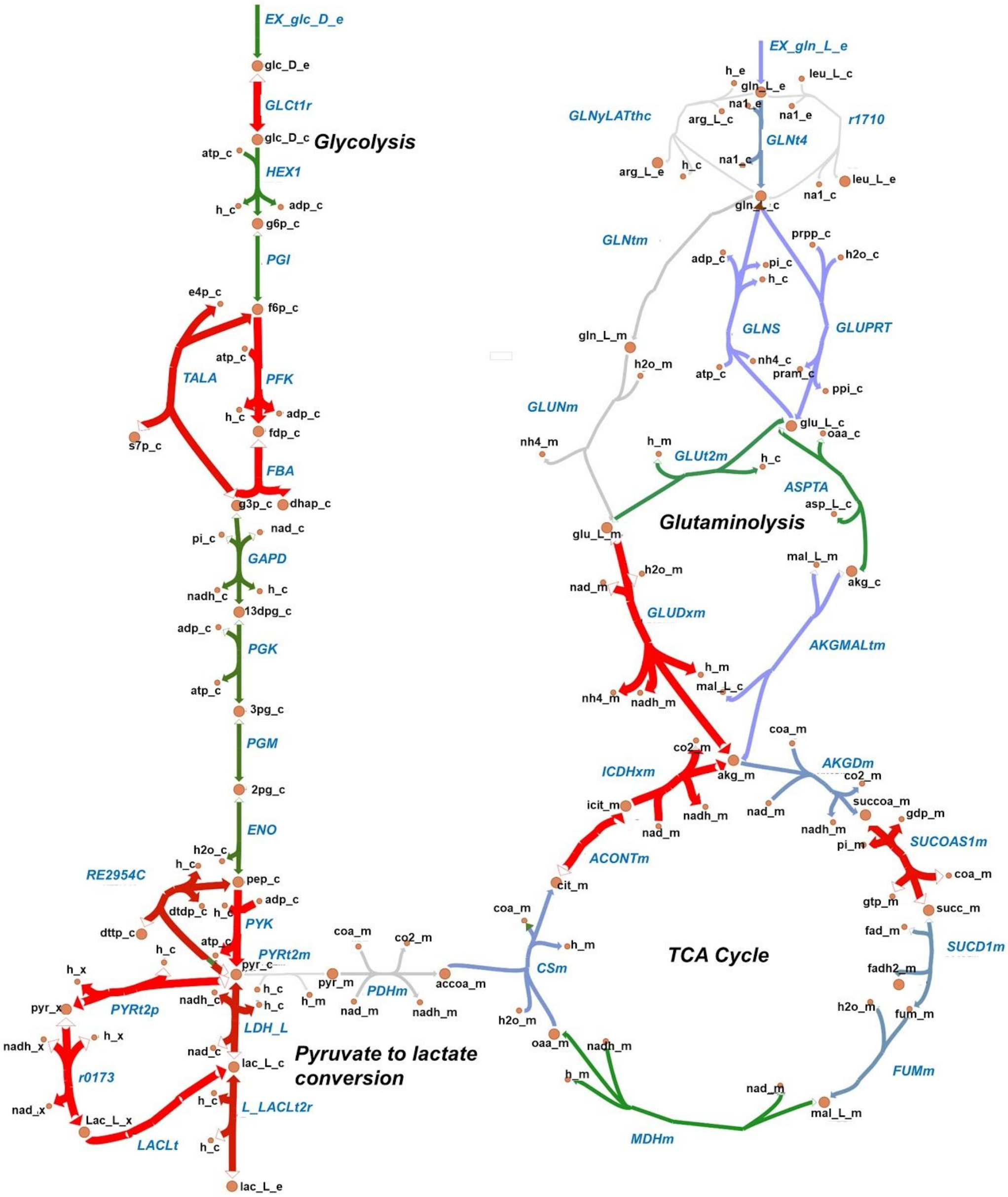

Supplementary Figure 4: Flux maps of metabolic pathways active in Th2 metabolic models

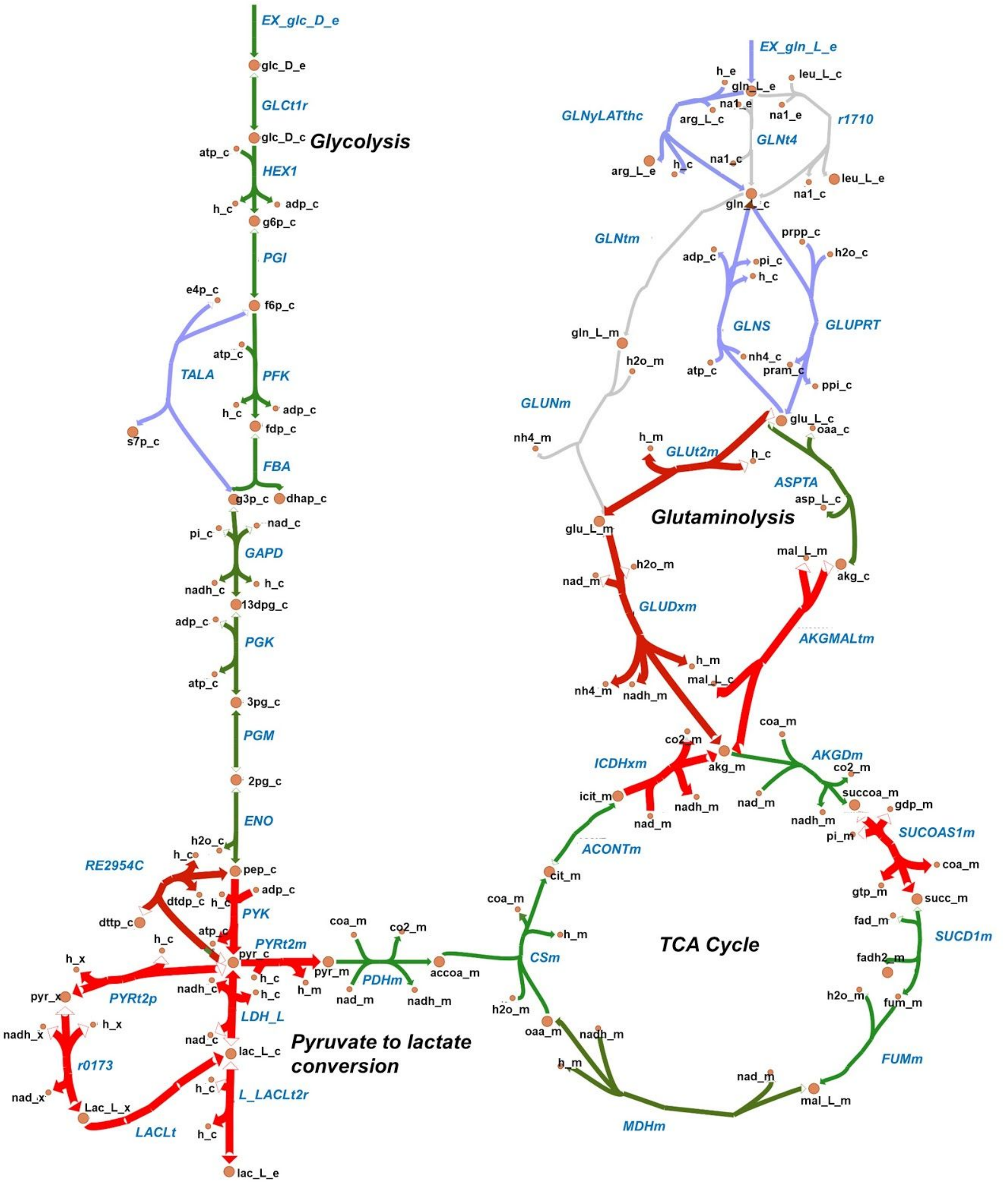

Supplementary Figure 5: Flux maps of metabolic pathways active in Th2 metabolic models

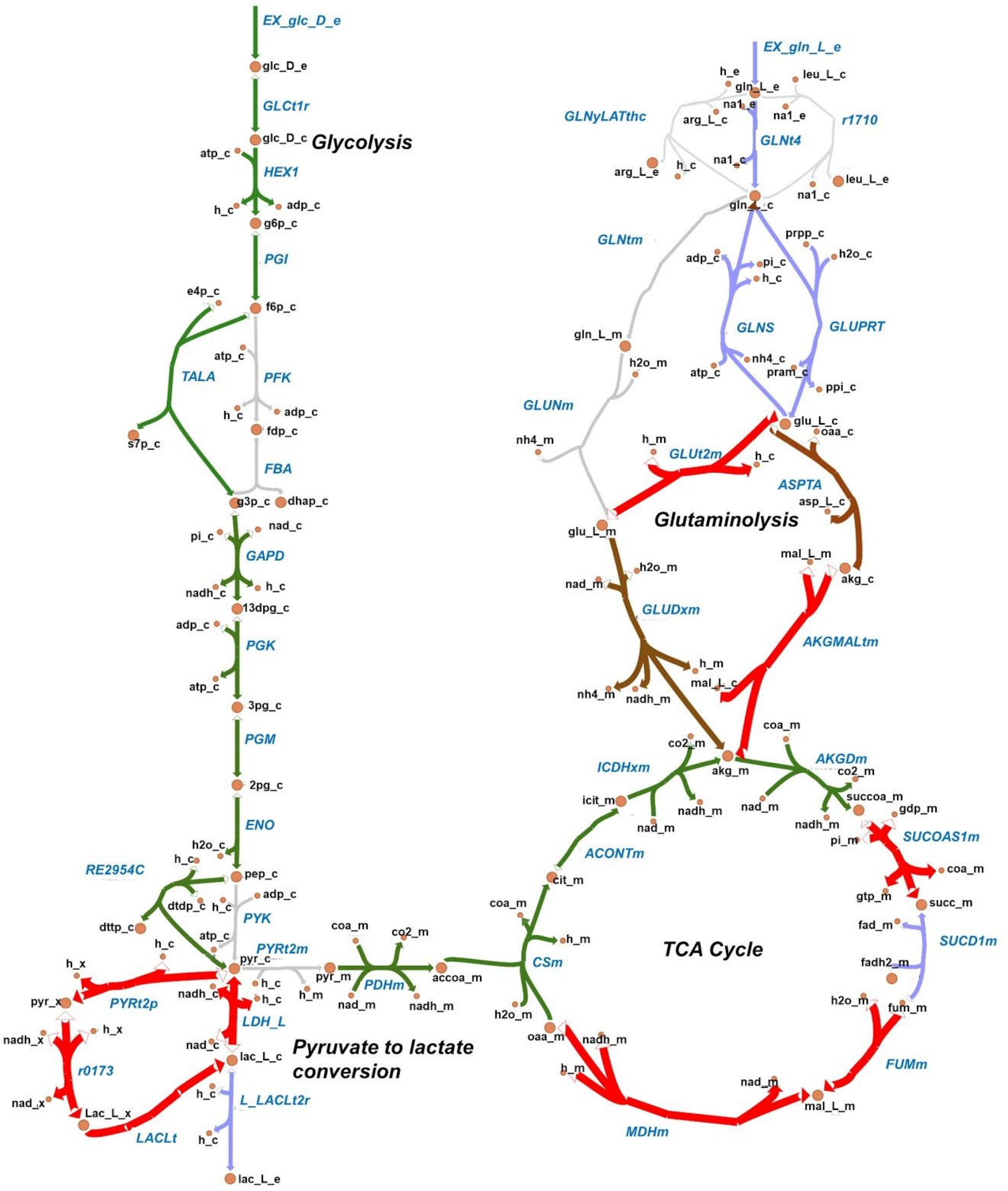

Supplementary Figure 6: Dependency of growth rate on Glucose in CD4T1670 model

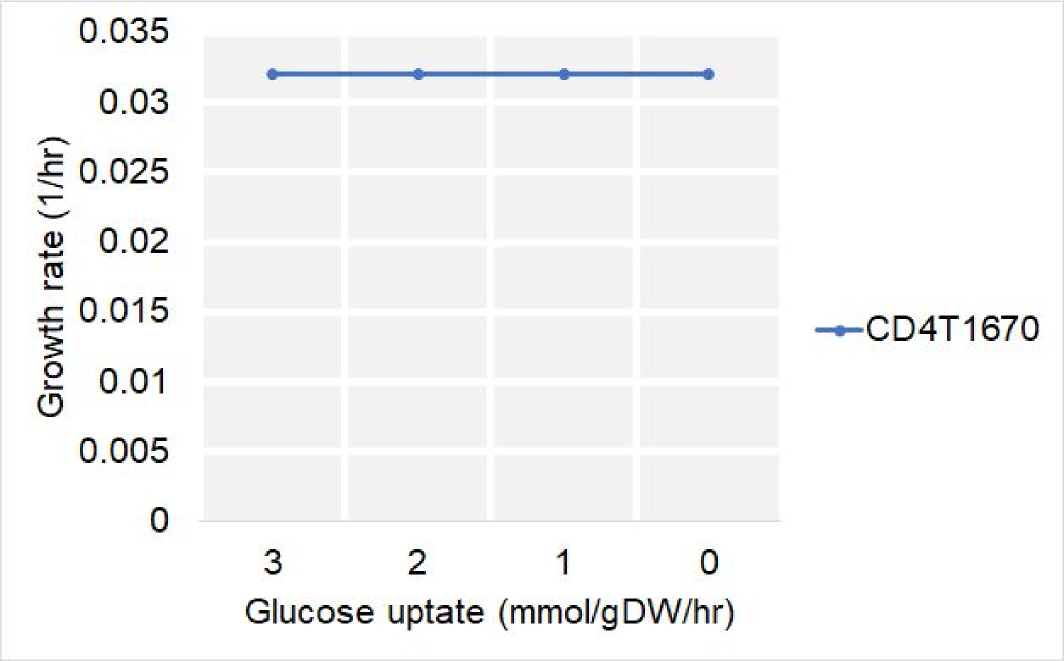

Supplementary Figure 7: Lactate production by all the models

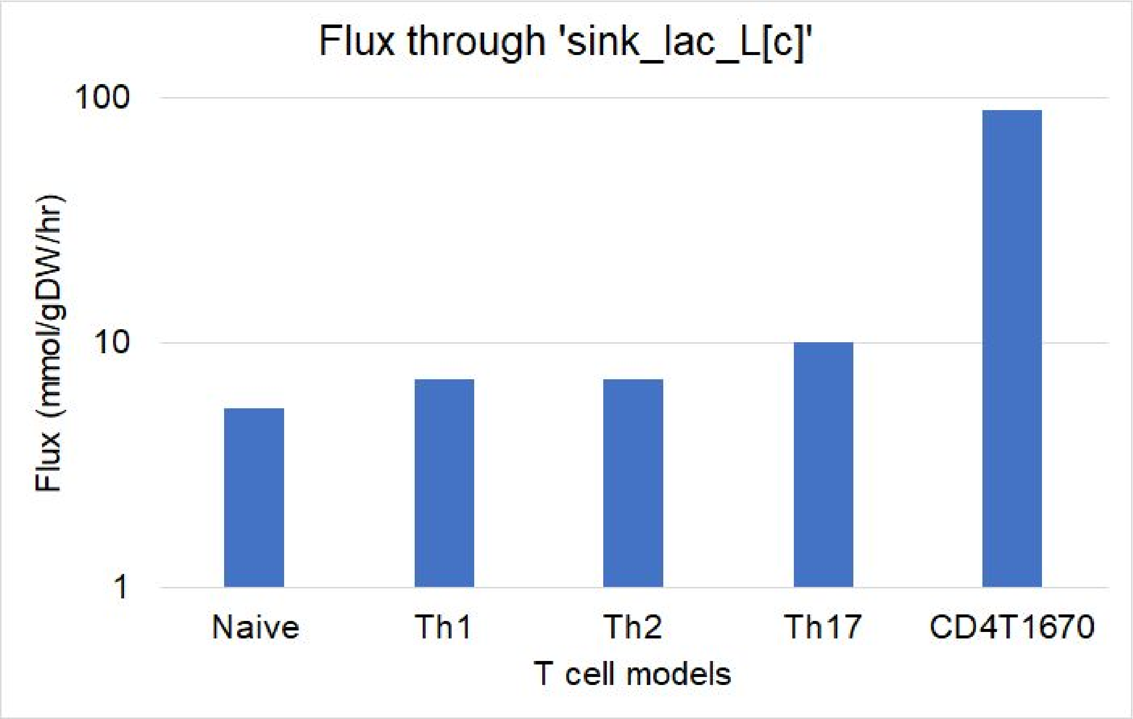

Supplementary Figure 8: Dependency of growth rate on glutamine in CD4T1670 model when glucose was removed.

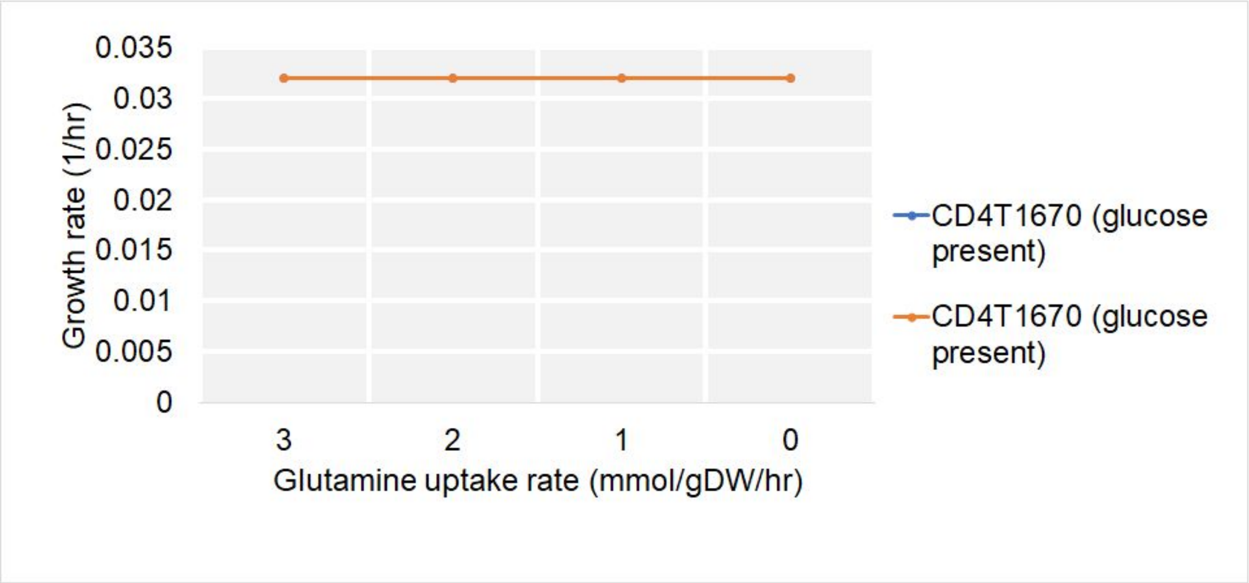

Supplementary Figure 9: Accuracy and precision based on comparison between essential genes predicted by models and experimentally defined

a

**Naive**		OGEE	
		Essential	Not essential
MODEL	Essential	62 (TP)	22 (FP)
	Not essential	392(FN)	579(TN)

b

**Th1**		OGEE	
		Essential	Not essential
MODEL	Essential	69 (TP)	26 (FP)
	Not essential	428 (FN)	610 (TN)

c

**Th2**		OGEE	
		Essential	Not essential
MODEL	Essential	61 (TP)	20 (FP)
	Not essential	421 (FN)	625 (TN)

d

**Th17**		OGEE	
		Essential	Not essential
MODEL	Essential	63 (TP)	21 (FP)
	Not essential	447(FN)	719(TN)

e

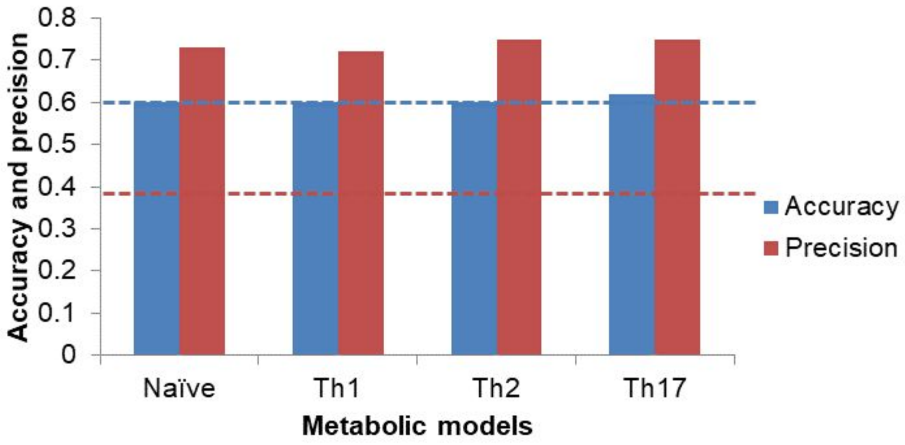

(a-d) Contingency tables for all the models. Rows: model predicted gene essentiality and columns: experimentally defined essentiality obtained from the OGEE database. (e) Accuracy and precision of all T cell subtype models based on the contingency table given in (a – d). Dotted horizontal lines show accuracy (blue) and precision (red) with randomly picked essential and non-essential genes from a population of 1892 (total metabolic genes in the reference model). The random accuracy and precision were obtained by averaging 100 samples.

Supplementary Figure 10: Precision-recall curve of higher-ranked genes based effect on growth after inhibition (Naïve).

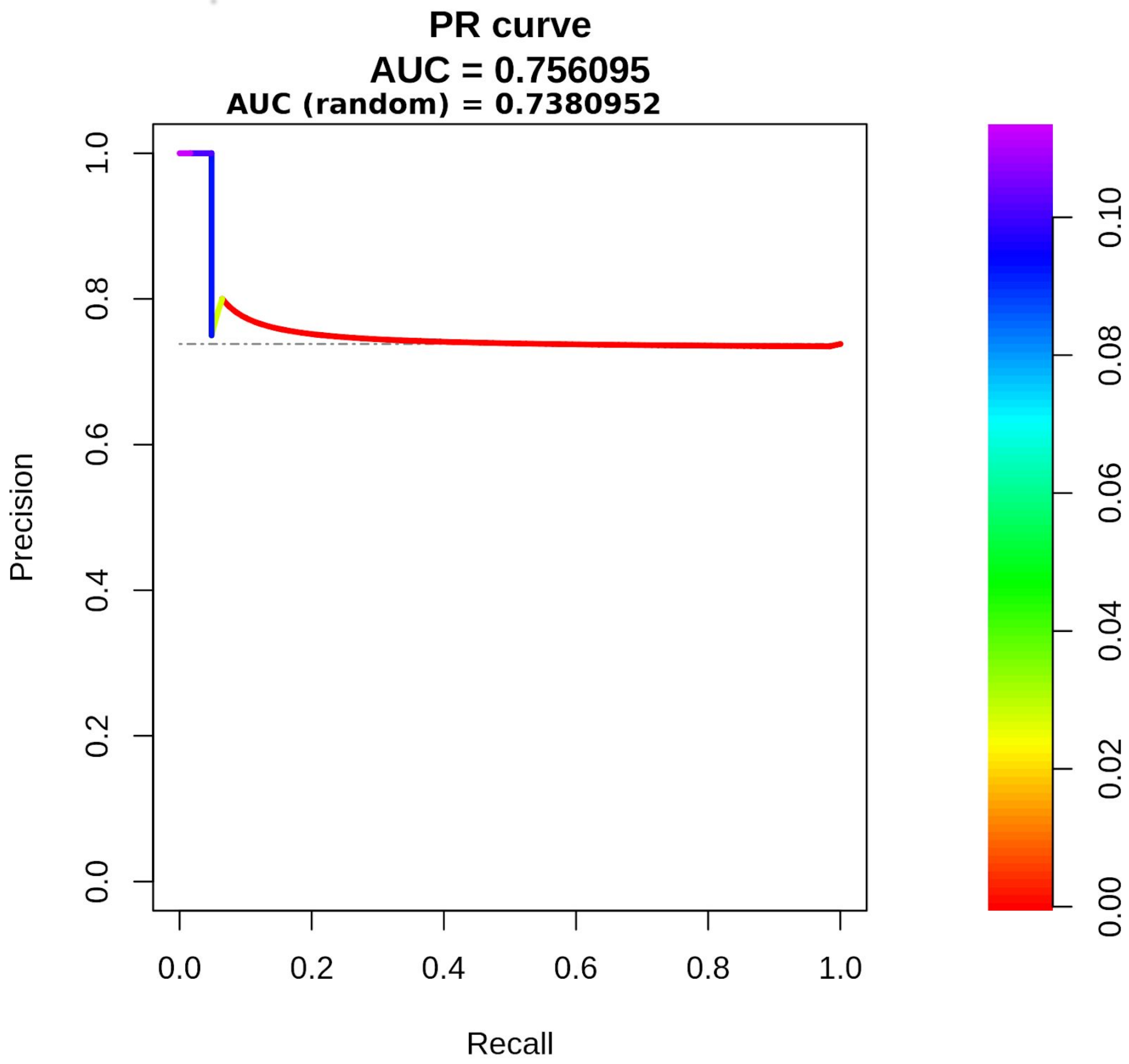

The curve was generated using top-ranked genes based on the effect on the growth rate. A total of 84 genes were included in the analysis. The horizontal dotted line shows the precision of a random classifier.

Supplementary Figure 11: Precision-recall curve of higher-ranked genes based effect on growth after inhibition (Th1).

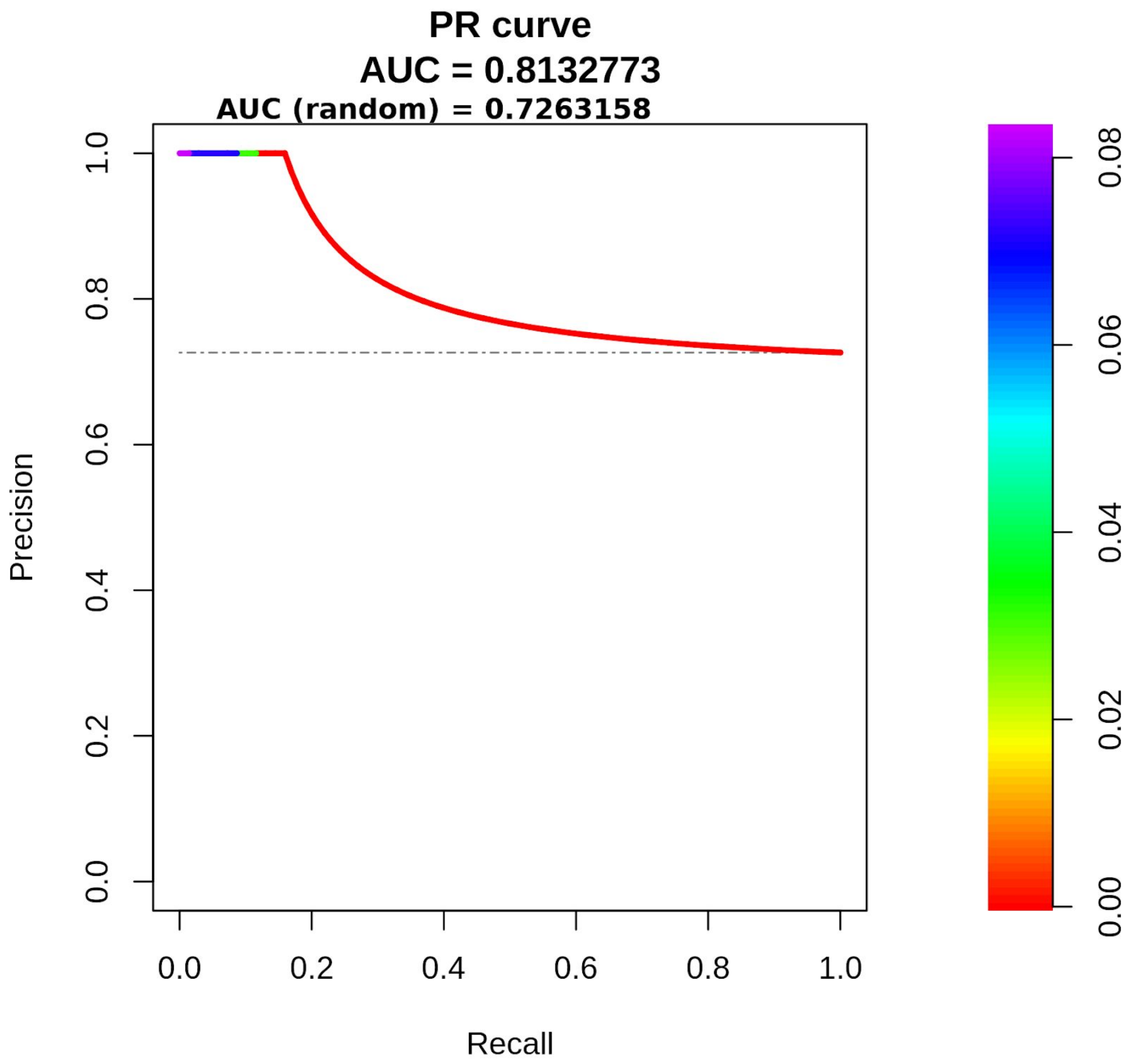

The curve was generated using top-ranked genes based on the effect on the growth rate. A total of 95 genes were included in the analysis. The horizontal dotted line shows the precision of a random classifier.

Supplementary Figure 12: Precision-recall curve of higher-ranked genes based effect on growth after inhibition (Th2).

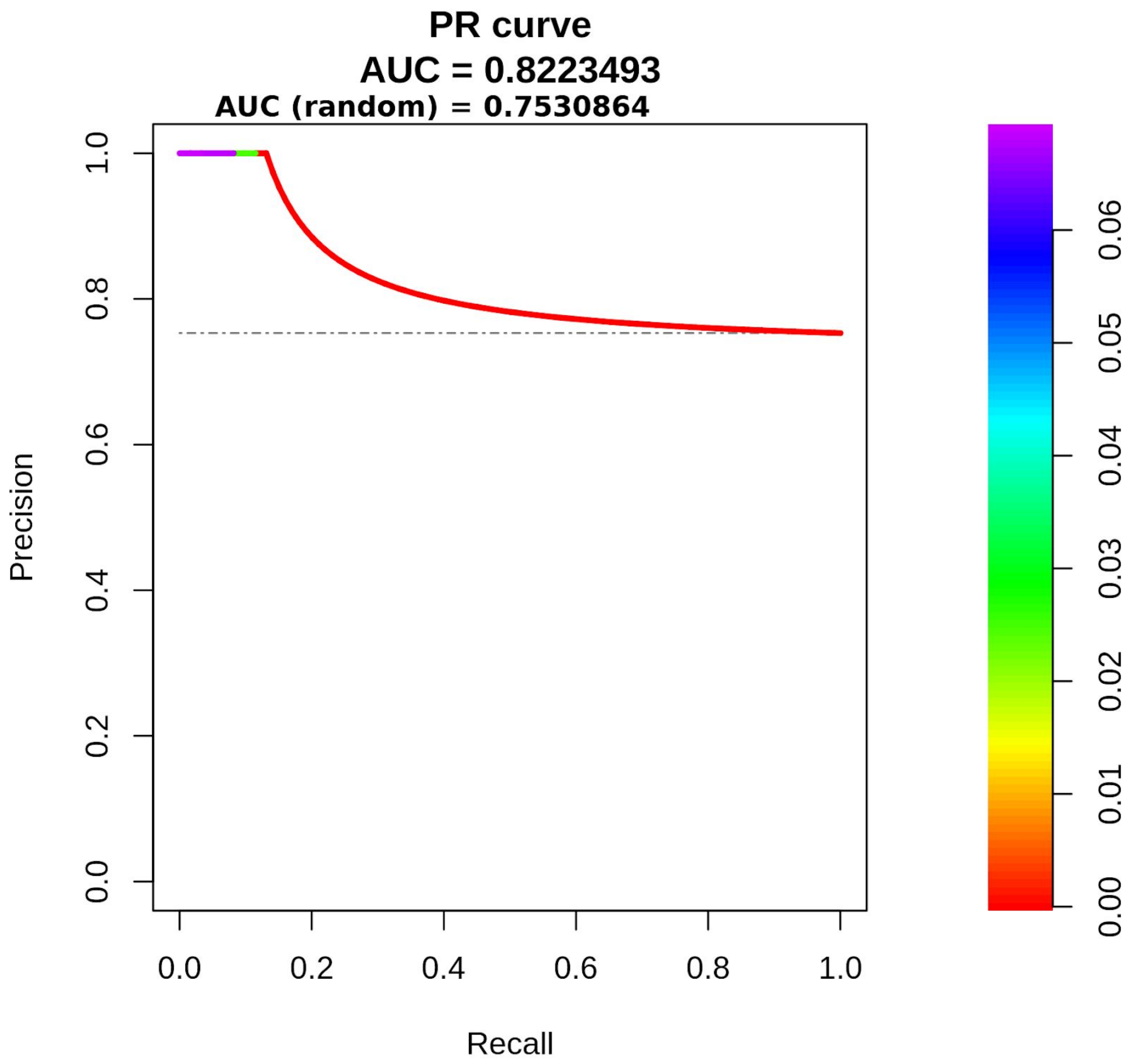

The curve was generated using top-ranked genes based on the effect on the growth rate. A total of 81 genes were included in the analysis. The horizontal dotted line shows the precision of a random classifier.

Supplementary Figure 13: Precision-recall curve of higher-ranked genes based effect on growth after inhibition (Th17).

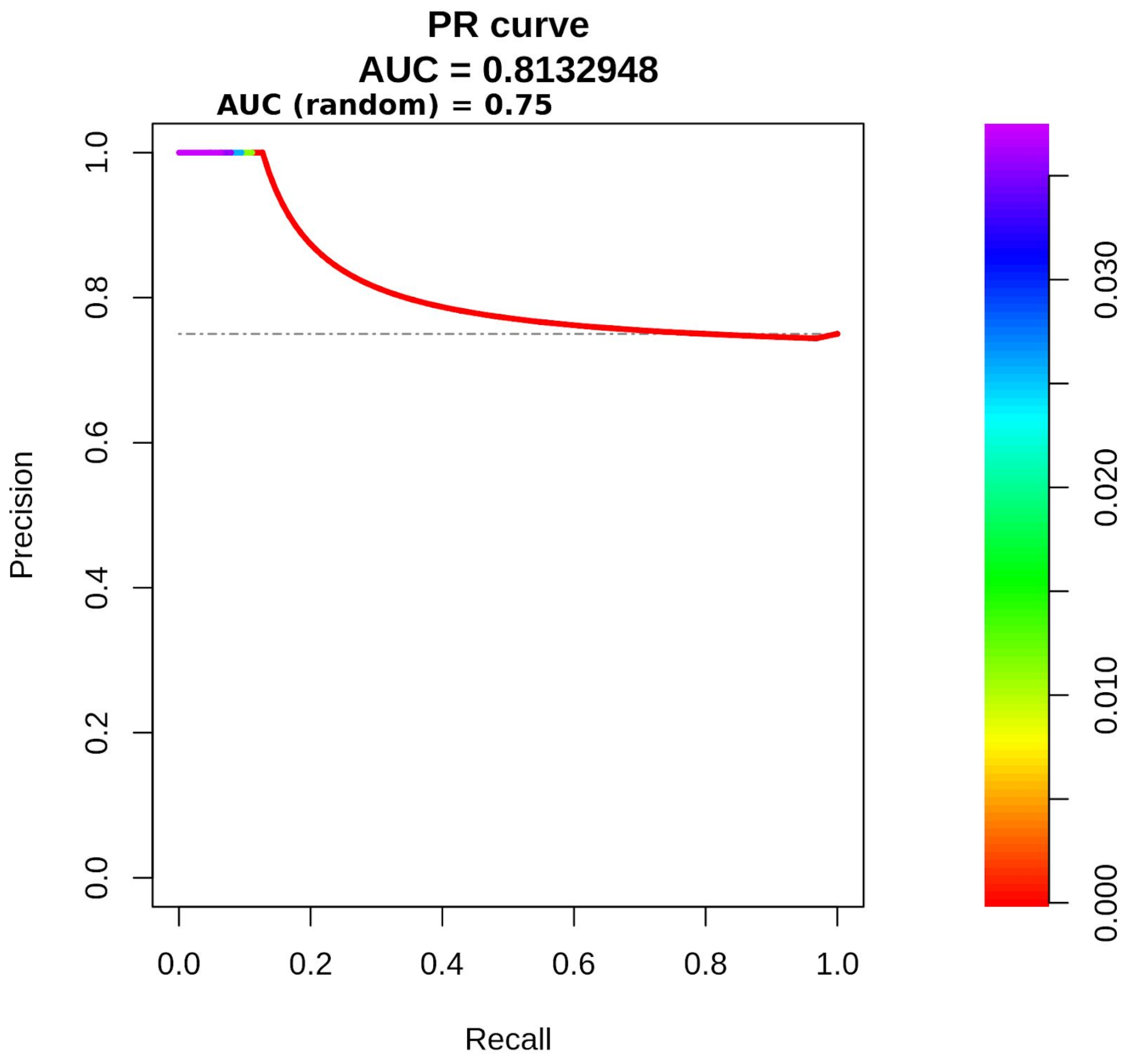

The curve was generated using top-ranked genes based on the effect on the growth rate. A total of 84 genes were included in the analysis. The horizontal dotted line shows the precision of a random classifier.

Supplementary Figure 14: Distribution of PES and their Z-scores in different models

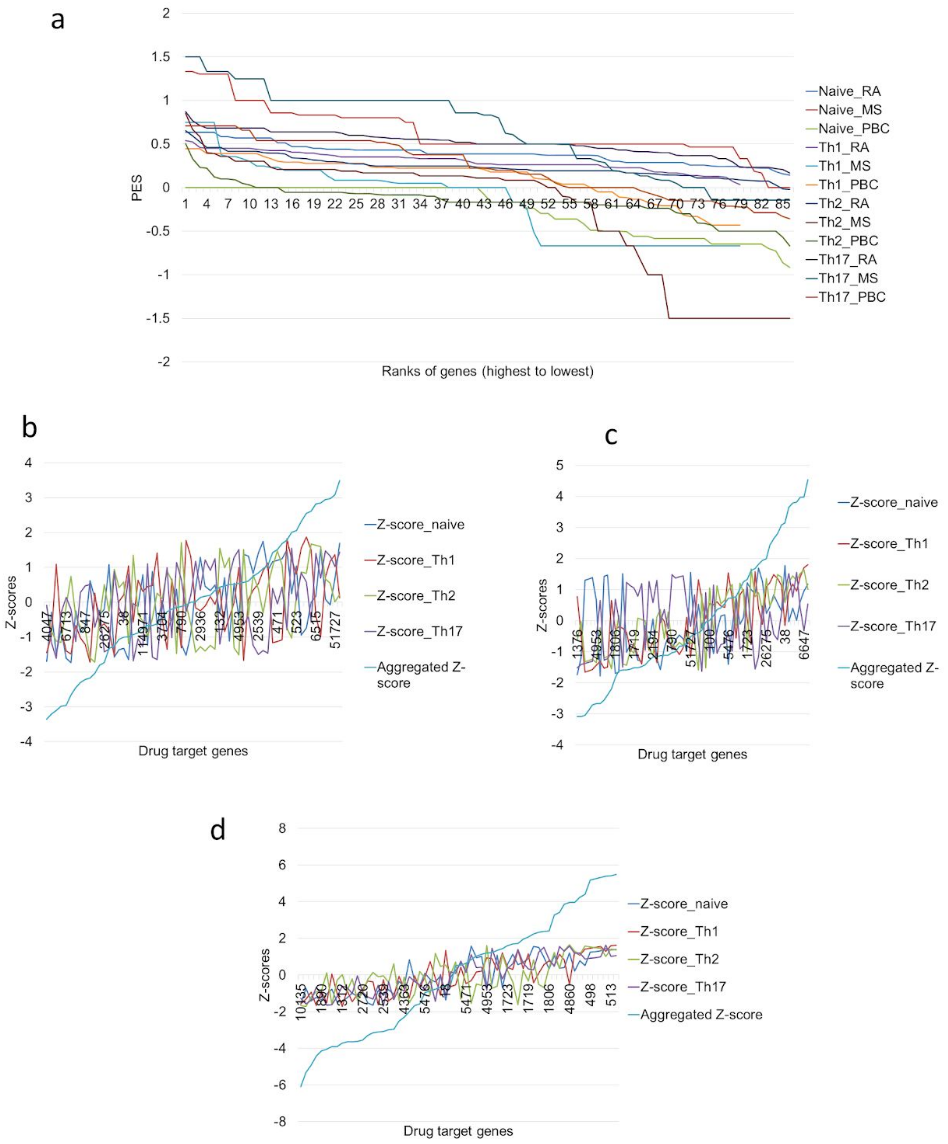

(a) Distribution of PES score in all 12 combinations of disease and CD4+ T cell. (d, e, and f) Z-scores based on PES of 62 genes that were common drug targets among naïve, Th1, Th2, and Th17 for (d) RA, (e) MS, and (f) PBC. In these plots, blue points represent aggregate Z-score, which is the raw sum of Z-scores across four CD4+ T cell subtypes.

Supplementary Figure 15: CD4+ T cell proliferation response upon drug treatment

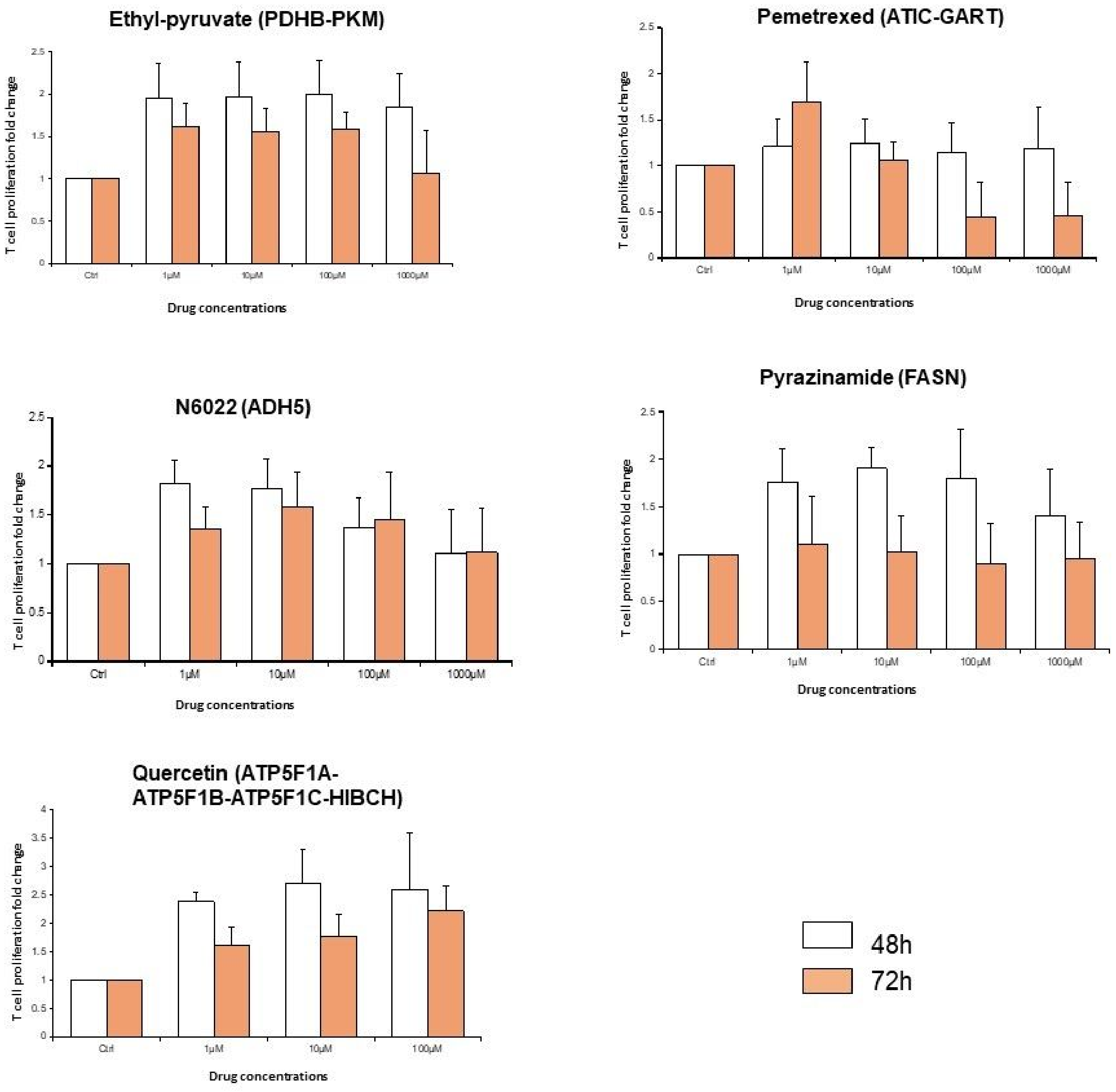

CD4+ T cells were exposed to various concentrations of drugs (1 µM, 10 µM, 100 µM, and 1000 µM) for 48 h (white bars) and 72 h (orange bars). Drugs' names are indicated on the top of each graph bar with their corresponding targeted gene in parentheses. CD4+ T Cell proliferation is expressed as fold change +/- SEM relative to untreated control cells (n = 4).

Supplementary Figure 16: Models constructed based on different datasets: transcriptomics, proteomics, and integrated

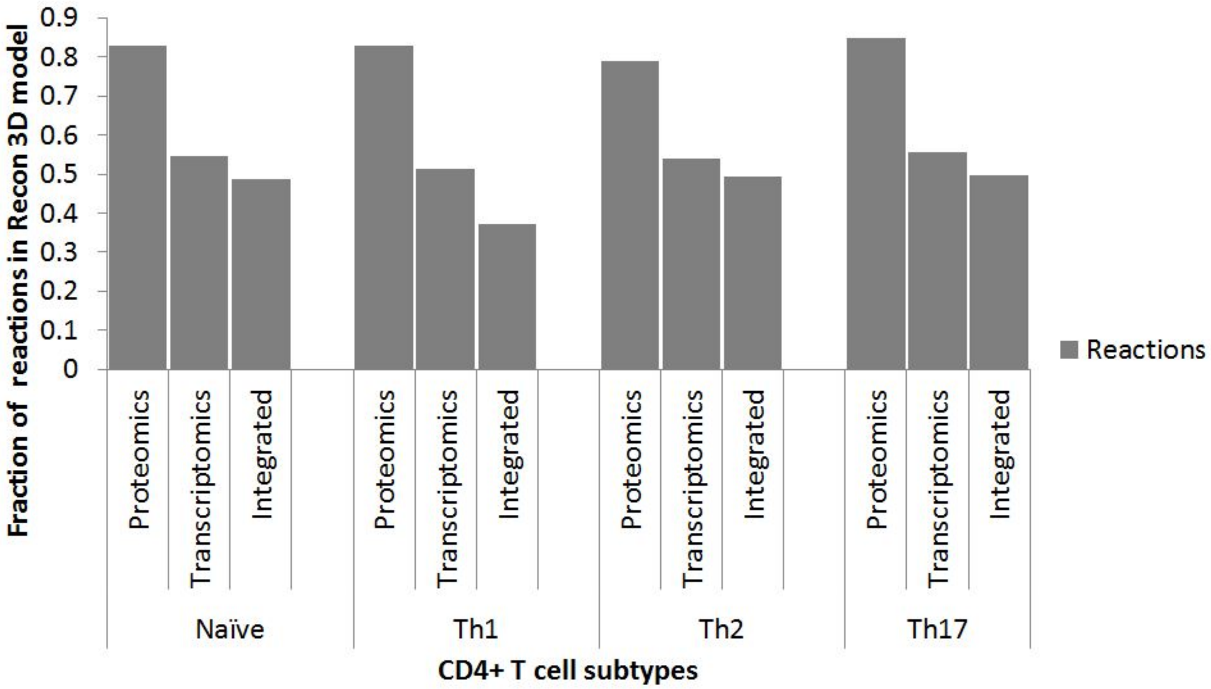

The number of reactions in constructed CD4+ T cell-specific models based on proteomics data, transcriptomics, and integrated (multi-omics) datasets. The bars show the fraction of reactions when compared to reactions in the modified Recon 3D model. The models based on proteomics data retained > 80% reactions of the modified Recon 3D. Transcriptomics-based models are significantly reduced and retained ~ 55% reactions of the modified Recon 3D. Integrated multi-omics based models have the lowest number of reactions among the three.

Supplementary Figure 17: Models constructed based on biomass reaction of iAB-AMO-1410 and Recon 3D

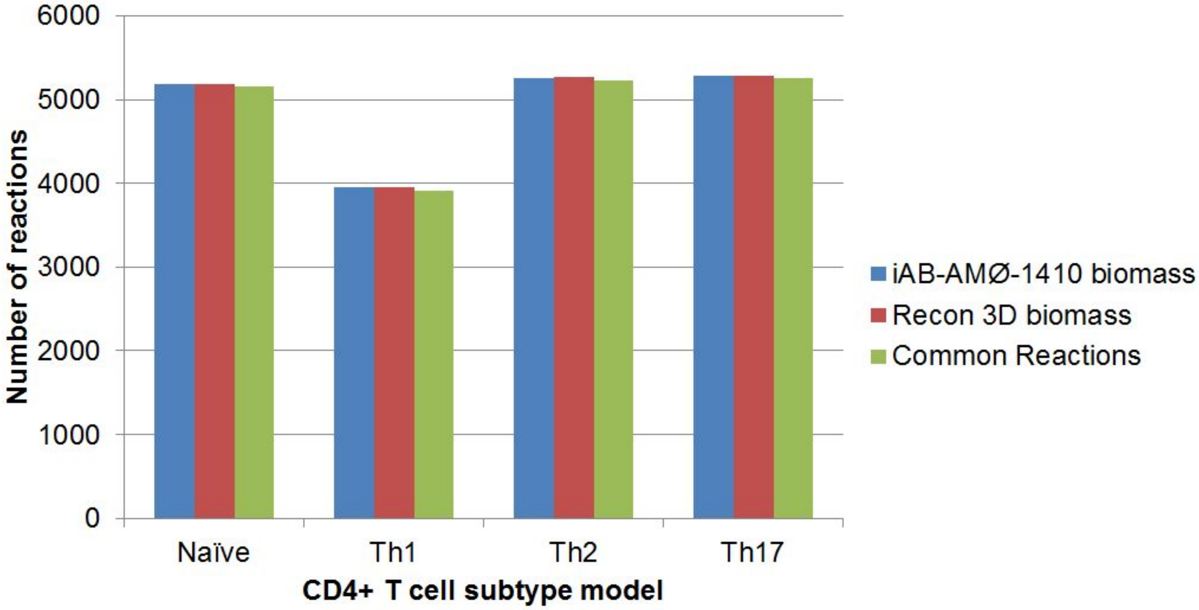

The number of reactions in the model constructed based on the iAB-AMO-1410 biomass equation and Recon3D biomass equation as well as common reactions between the two. Our results indicate that changing biomass function has minimal effect on the models. A total of >98% of reactions are overlapped for all the models.
